# Plant-Derived Whole Foods and Food-Based Supplements on Post-Exercise Recovery in Physically Active Individuals: A Systematic Review of Human Intervention Trials

**DOI:** 10.3390/nu18183049

**Published:** 2026-09-18

**Authors:** Dang Hien Ngan Nguyen, Tsung-Wei Yu, Quan Buu Quach, Abinaya Raja, Chiao-Ming Chen, Margaret Jip Kuo, Thanh Nam Truong, Sing-Chung Li

**Affiliations:** 1 Department of Nutrition and Food Safety, Faculty of Public Health, Can Tho University of Medicine and Pharmacy, Can Tho 94000, Vietnam; ndhngan@ctump.edu.vn (D.H.N.N.); ttnam@ctump.edu.vn (T.N.T.); 2 Department of Medical Education and Research, Kaohsiung Veterans General Hospital, Kaohsiung City 81346, Taiwan; willy890317@gmail.com; 3 Medical Education and Research Office, Kaohsiung Armed Forces General Hospital, Kaohsiung City 802301, Taiwan; 4 Department of Geriatrics and Gerontology, University of Medicine and Pharmacy at Ho Chi Minh City, Ho Chi Minh City 700000, Vietnam; drquanbuu25@gmail.com; 5 Graduate Institute of Medical Genomics and Proteomics, College of Medicine, National Taiwan University, Taipei 100225, Taiwan; abinayaraja2712@gmail.com; 6 Department of Food Science, Nutrition, and Nutraceutical Biotechnology, Shih Chien University, Taipei 10462, Taiwan; charming@g2.usc.edu.tw; 7 Sports Medicine Division, Hong Kong Sports Institute Limited, 25 Yuen Wo Road, Sha Tin, New Territories, Hong Kong SAR, China; margaretk@hksi.org.hk; 8 School of Nutrition and Health Sciences, College of Nutrition, Taipei Medical University, Taipei 11031, Taiwan

**Keywords:** plant-derived whole foods, food-first sports nutrition, exercise recovery, physically active individuals, muscle soreness

## Abstract

Background/Objectives: Post-exercise recovery is essential for maintaining training quality and subsequent performance. Plant-derived whole foods and food-based supplements may support recovery through antioxidant, anti-inflammatory, vascular, and metabolic mechanisms; however, the available evidence remains heterogeneous. This systematic review evaluated their effects on muscle soreness, muscle-function recovery, physical performance, and physiological responses in physically active individuals. Methods: PubMed, Scopus, Embase, and Web of Science were searched for English-language human intervention studies published between 2016 and 2026. Eligible studies included physically active participants aged ≥ 14 years receiving plant-derived whole foods or food-based supplements, with outcomes related to post-exercise recovery. Findings were synthesized narratively because substantial heterogeneity in participants, exercise protocols, interventions, doses, timing, comparators, and outcome measures precluded meta-analysis. Risk of bias was assessed using the Cochrane RoB 2 tool for randomized trials and ROBINS-I for the non-randomized intervention study. Results: Thirty-six human intervention studies were included. Interventions comprised polyphenol-rich products such as tart cherry, pomegranate, berries, grape, and anthocyanin-rich juices; nitrate- or citrulline-rich foods such as beetroot and watermelon; and other plant-derived products including banana, honey, mango, chicory, and oat-based foods. Reductions in perceived muscle soreness or delayed-onset muscle soreness were reported in several studies, particularly within 24–72 h after exercise; however, these findings were not uniform across interventions and study designs. Considerable variation in product form, bioactive composition, dose, supplementation duration, timing, dietary control, and adverse-event reporting limited direct comparisons. Among the 35 randomized trials assessed using RoB 2, 11 were judged to have a low overall risk of bias and 24 raised some concerns; none was judged to have a high overall risk of bias. The non-randomized study was assessed separately using ROBINS-I and was judged to have a serious overall risk of bias. Conclusions: Plant-derived whole foods and food-based supplements may support selected aspects of post-exercise recovery, particularly perceived muscle soreness. However, current evidence does not establish consistent benefits for muscle-function recovery, subsequent performance, or biochemical outcomes, and no specific intervention, dose, or timing strategy can yet be recommended based on the currently available evidence.

## 1. Introduction

Post-exercise recovery is a central component of sports nutrition because it influences subsequent training quality, performance readiness, and long-term adaptation [1,2]. Sustained exercise participation is also shaped by motivational factors, including perceived health benefits, supportive exercise environments, life goals, and exercise enjoyment [3]. Strenuous exercise, especially exercise involving repeated eccentric muscle actions, can cause exercise-induced muscle damage (EIMD). This condition commonly occurs after activities such as resistance exercise, downhill running, sprinting, jumping, or prolonged endurance exercise. EIMD is usually followed by delayed-onset muscle soreness (DOMS), temporary loss of muscle strength, reduced range of motion, and impaired physical performance [4,5]. These symptoms may persist for several days, thereby reducing training quality, delaying return to exercise, and compromising subsequent performance.

EIMD generally develops through two overlapping phases. The primary phase involves mechanical disruption of muscle fibers and sarcomeres, whereas the secondary phase is characterized by inflammatory responses and increased production of reactive oxygen species (ROS) [5,6]. This secondary response may persist or peak between 48 and 96 h after exercise, although many studies assess recovery only within the first 48 h. Therefore, nutritional strategies that alleviate muscle soreness, promote the restoration of muscle function, and modulate post-exercise inflammation and oxidative stress are of considerable practical importance for physically active individuals [7,8].

Sports nutrition has increasingly shifted toward a “food-first” approach, emphasizing the use of whole foods and food-based products before isolated or synthetic supplements [9]. Within this context, plant-derived whole foods and minimally processed food-based supplements are of particular interest because they provide complex food matrices containing multiple nutrients and bioactive compounds. These include polyphenols, anthocyanins, flavonoids, betalains, dietary nitrates, citrulline, carbohydrates, vitamins, and minerals. Moreover, the growing adoption of vegetarian and vegan dietary patterns increases the practical need for evidence-based post-exercise recovery strategies that are compatible with plant-based dietary preferences [10]. Whole or minimally processed plant foods may also be more consistent with a food-first strategy because they retain complex food matrices and provide multiple nutrients and bioactive compounds rather than a single isolated constituent. Plant-derived foods such as tart cherry, beetroot, pomegranate, grape, berries, watermelon, banana, and other fruit- or vegetable-derived products provide a complex food matrix containing multiple bioactive compounds [11]. These include polyphenols, anthocyanins, flavonoids, betalains, dietary nitrates, citrulline, carbohydrates, vitamins, minerals, and other antioxidant or anti-inflammatory constituents [12]. Unlike single-compound supplements, plant-derived food interventions may act through overlapping and complementary mechanisms, including modulation of inflammatory signaling [13], attenuation of oxidative stress [14], enhancement of nitric oxide bioavailability [15], improved endothelial function and blood flow, support of substrate availability, and regulation of post-exercise metabolic responses. The food matrix can influence the release, stability, bioaccessibility, absorption, metabolism, and ultimately the biological activity of phytochemicals. Depending on interactions with proteins, lipids, carbohydrates, dietary fiber, and processing conditions, these effects may either enhance or reduce their systemic bioavailability [16].

Several plant-derived interventions have been investigated for post-exercise recovery, particularly tart cherry, beetroot juice, pomegranate, grape products, and berry-based foods or supplements. However, the available evidence remains heterogeneous and difficult to interpret. Previous systematic reviews and meta-analyses have often focused on individual food sources, specific classes of polyphenols, or isolated extracts, with many emphasizing outcomes assessed within the first 24–48 h after exercise [17]. This time frame may not fully capture the recovery process because muscle soreness, impaired muscle function, and biochemical markers of muscle damage may peak or remain altered beyond 48 h and, in some cases, persist for up to 72–96 h, depending on the exercise protocol and participants’ training status. Moreover, previous reviews have not always clearly distinguished whole foods and food-based supplements from purified phytochemical extracts or single-nutrient products. Interpretation is further complicated by substantial variation in exercise modality, participant characteristics, intervention form, dose, timing, duration, comparator conditions, outcome definitions, and follow-up schedules.

Accordingly, this systematic review aims to synthesize and critically evaluate the effects of plant-derived whole foods and food-based supplements on post-exercise recovery in physically active individuals. The review prioritizes direct recovery outcomes, including muscle soreness/DOMS and restoration of muscle function, assessed using measures such as maximal voluntary contraction, maximal isometric voluntary contraction, countermovement jump, vertical jump, range of motion, wall-sit performance, and sprint or agility recovery. Secondary outcomes include biomarkers of muscle damage, inflammation, oxidative stress, antioxidant status, metabolic and cardiovascular responses, and other relevant physiological measures. By organizing the evidence according to intervention type, recovery-outcome domain, and proposed biological mechanisms, this review seeks to provide a practical and mechanistically informed framework for sports nutrition researchers, practitioners, and physically active populations.

## 2. Materials and Methods

### 2.1. Protocol and Registration

This systematic review was conducted in accordance with the Preferred Reporting Items for Systematic Reviews and Meta-Analyses (PRISMA 2020) guidelines to ensure methodological transparency and reproducibility. The protocol for this review was prospectively registered in the International Prospective Register of Systematic Reviews (PROSPERO; registration ID: CRD420261369778). The primary objective of this review was to systematically evaluate the effects of plant-derived whole food interventions on exercise recovery outcomes in physically active individuals compared with placebo, habitual diet, or no intervention. The completed PRISMA 2020 checklist is provided in Supplementary Materials.

### 2.2. Eligibility Criteria

Study eligibility was defined a priori according to the PICOS framework, including population, intervention, comparator, outcomes, and study design. Eligible studies were peer-reviewed original research articles published in English between 2016 and 2026. This publication window was prespecified in the review protocol to focus the synthesis on contemporary evidence concerning plant-derived food-based interventions and current sports-nutrition practices. Nevertheless, we recognize that this restriction may have excluded relevant studies published before 2016. The population of interest comprised healthy human participants aged ≥ 14 years, including adolescent and adult individuals, who were physically active, recreationally trained, trained, or competitive athletes. Eligible interventions included plant-derived whole foods and food-based supplements consumed in relation to exercise recovery, such as juices, concentrates, freeze-dried powders, purees, beverages, or whole-food snacks. Studies were excluded when the intervention consisted solely of isolated synthetic compounds, purified phytochemical extracts without a clearly identifiable food source, or multi-ingredient formulations in which the independent effect of the plant-derived component could not be determined. Eligible comparator conditions included placebo, isocaloric control, habitual diet, or no-intervention control. Studies were required to report at least one review-relevant outcome, including either a primary recovery outcome, such as muscle soreness/DOMS or restoration of muscle function, or a secondary biochemical or physiological marker related to muscle damage, inflammation, oxidative stress, metabolic recovery, cardiovascular response, hydration status, or other recovery-related processes. Eligible study designs included randomized controlled trials, randomized crossover trials, and controlled clinical trials.

For the purposes of this review, a food-based supplement was operationally defined as a preparation derived from an identifiable edible plant food that retained a multi-component food-derived matrix or naturally occurring mixture of constituents, despite processing such as juicing, concentration, drying, freeze-drying, pureeing, or incorporation into a food or capsule-based preparation. Preparations consisting primarily of an isolated or purified single phytochemical, synthetic compound, or highly purified botanical constituent without a retained food-derived matrix were excluded. Multi-ingredient formulations were excluded when the independent contribution of the eligible plant-derived food component could not be determined.

While the formal systematic review synthesis was restricted to human intervention trials, preclinical studies (cell-based and animal models) were selectively identified from the literature to provide supporting evidence for biological mechanisms. These studies were used solely for mechanistic interpretation and were not included in the PRISMA search statistics, formal data extraction for efficacy, risk-of-bias assessment, or qualitative synthesis of primary outcomes.

### 2.3. Information Sources and Search Strategy

A comprehensive literature search was conducted in PubMed, Scopus, Embase, and Web of Science to identify relevant studies. Following peer-review comments, the search strategy was revised to improve sensitivity by broadening the intervention terminology and incorporating general terms for plant-derived foods and food-based interventions in addition to specific food terms. The updated searches were conducted in PubMed, Scopus, and Embase on 21 August 2026. These searches identified 805 records from PubMed, 1014 from Scopus, and 750 from Embase, yielding a total of 2569 records before deduplication. Web of Science could not be rerun during the search-update period because database access was unavailable.

The search strategy combined terms related to three main concepts: plant-derived interventions (e.g., plant-derived, plant-based, plant food, whole food, fruit, vegetable, juice, concentrate, powder, tart cherry, beetroot, pomegranate, grape, berry, honey, mango, and chicory); exercise and exercise-related conditions; and post-exercise recovery outcomes, including muscle soreness, muscle function, strength recovery, performance recovery, inflammation, and oxidative stress. Database-specific controlled vocabulary, including relevant MeSH terms in PubMed, was combined with free-text terms where appropriate. Search syntax was adapted for each database. Reference lists of eligible articles and relevant reviews were also screened to identify potentially eligible studies. The complete database-specific search strategies, search dates, and retrieval counts are provided in Supplementary Table S1.

### 2.4. Study Selection Process

After duplicate removal, two reviewers independently screened the titles and abstracts of all retrieved records according to the predefined eligibility criteria. Potentially eligible reports were subsequently retrieved and assessed in full text by the same reviewers. Disagreements at either stage were resolved through discussion and, when necessary, consultation with a third reviewer. Reasons for full-text exclusion were recorded and summarized in the PRISMA flow diagram. When multiple reports described the same participant sample or trial, they were linked and treated as a single study unless they represented distinct intervention protocols, exercise conditions, or participant cohorts.

### 2.5. Data Extraction and Items

Data were extracted independently by two reviewers using a standardized data extraction form. Extracted information included author and year of publication, country, study design, participant characteristics, sample size, age, training status, exercise protocol, intervention characteristics, comparator condition, timing and duration of supplementation, outcome measures, and main findings. Where reported and applicable, numerical outcome data, measures of variability, effect estimates, and between-group or between-condition differences were also extracted. The standardized extraction form was not formally piloted on a prespecified subset of studies before full data extraction. Any discrepancies in data extraction were resolved through discussion or consultation with a third reviewer when necessary.

To ensure consistency across studies, outcomes were classified into four predefined domains. The first domain was muscle soreness/DOMS, which included subjective soreness or pain ratings assessed using visual analogue scales (VAS), numeric or Likert-type scales, and objective pain-related measures such as pressure pain threshold. The second domain was muscle function recovery, defined as the restoration of force-generating capacity or functional performance after exercise, including maximal voluntary contraction, maximal isometric voluntary contraction, peak torque, countermovement jump, vertical jump, range of motion, wall-sit performance, handgrip strength, sprint/agility recovery, and related neuromuscular outcomes. The third domain was other physical performance, which included direct exercise capacity or performance tests, such as time to exhaustion (TTE), running economy, time trials, repeated sprint performance, or sport-specific performance tasks. The fourth domain was secondary physiological outcomes, including biomarkers of muscle damage (CK, LDH), inflammation (CRP, cytokines), oxidative stress, antioxidant status, metabolic responses, cardiovascular responses, and other recovery-related physiological measures.

To distinguish genuine post-exercise recovery from acute ergogenic effects, an outcome was classified as a recovery outcome when it was assessed following an initial exercise challenge and reflected the restoration or resolution of an exercise-induced disturbance during the subsequent recovery period. In contrast, performance or physiological outcomes measured during or immediately following an exercise bout after supplementation, without assessment of restoration from a preceding exercise-induced impairment, were classified as other physical performance or physiological outcomes, as appropriate, and were not interpreted as direct evidence of post-exercise recovery. Where this distinction was unclear because of the study design or timing of outcome assessment, the outcome was interpreted conservatively and was not considered direct evidence of recovery.

### 2.6. Risk of Bias Assessment

Risk of bias was assessed independently by two reviewers using design-appropriate assessment tools. Randomized controlled trials were evaluated using the revised Cochrane Risk of Bias tool for randomized trials (RoB 2), which assesses bias arising from the randomization process, deviations from intended interventions, missing outcome data, measurement of outcomes, and selection of the reported result. Each RoB 2 domain was judged as low risk of bias, some concerns, or high risk of bias.

The non-randomized intervention study (Ammar et al., 2016 [18]) was assessed separately using the Risk of Bias In Non-randomized Studies–of Interventions (ROBINS-I) tool. ROBINS-I evaluates bias due to confounding, selection of participants, classification of interventions, deviations from intended interventions, missing data, measurement of outcomes, and selection of the reported result. Domain-level and overall judgments were classified as low, moderate, serious, or critical risk of bias, or no information.

Any disagreements between reviewers were resolved through discussion and consensus, with consultation with a third reviewer when necessary. Risk-of-bias assessments were used to inform interpretation of the evidence and were not used as criteria for study exclusion. Results from RoB 2 and ROBINS-I were reported separately because the two tools use different domains and judgment categories.

### 2.7. Data Synthesis

The feasibility of meta-analysis was considered for clinically comparable intervention–outcome subsets. However, substantial clinical and methodological heterogeneity remained within potentially comparable subsets, including differences in participant characteristics, training status, exercise protocols, intervention formulation, supplementation dose, timing and duration, comparator conditions, outcome measures, and follow-up time points. The interventions also varied widely in food matrix, including whole fruits, fresh juices, purees, concentrates, freeze-dried powders, beverages, enriched food products, and capsule-based food preparations. This variability was considered important because the food matrix may influence the stability, bioavailability, and physiological activity of plant-derived bioactive compounds. Consequently, quantitative pooling was considered inappropriate, and a narrative synthesis was performed.

Studies were first grouped according to intervention category, including polyphenol-rich fruits, nitrate-rich foods, carbohydrate- and electrolyte-containing plant-based beverages, and other plant-derived food-based supplements. Within each category, results were further summarized according to outcome domain, including muscle soreness/DOMS, muscle function recovery, other physical performance outcomes, and secondary physiological outcomes. This approach allowed patterns in the direction and consistency of effects to be identified, while also considering plausible biological mechanisms across heterogeneous study designs.

The narrative synthesis considered the direction and magnitude of effects, measures of variability or precision, between-group or between-condition differences, and consistency of findings across studies, where these data were reported. Statistical significance was considered alongside these factors rather than being used as the sole basis for interpreting intervention effects.

Because the review was designed as a narrative synthesis across clinically and methodologically heterogeneous intervention–outcome bodies of evidence, a formal GRADE certainty assessment was not undertaken. Accordingly, no outcome-specific certainty ratings are reported, and conclusions are framed as a narrative synthesis rather than as graded estimates of certainty.

## 3. Results

### 3.1. Study Selection

**Figure 1:**
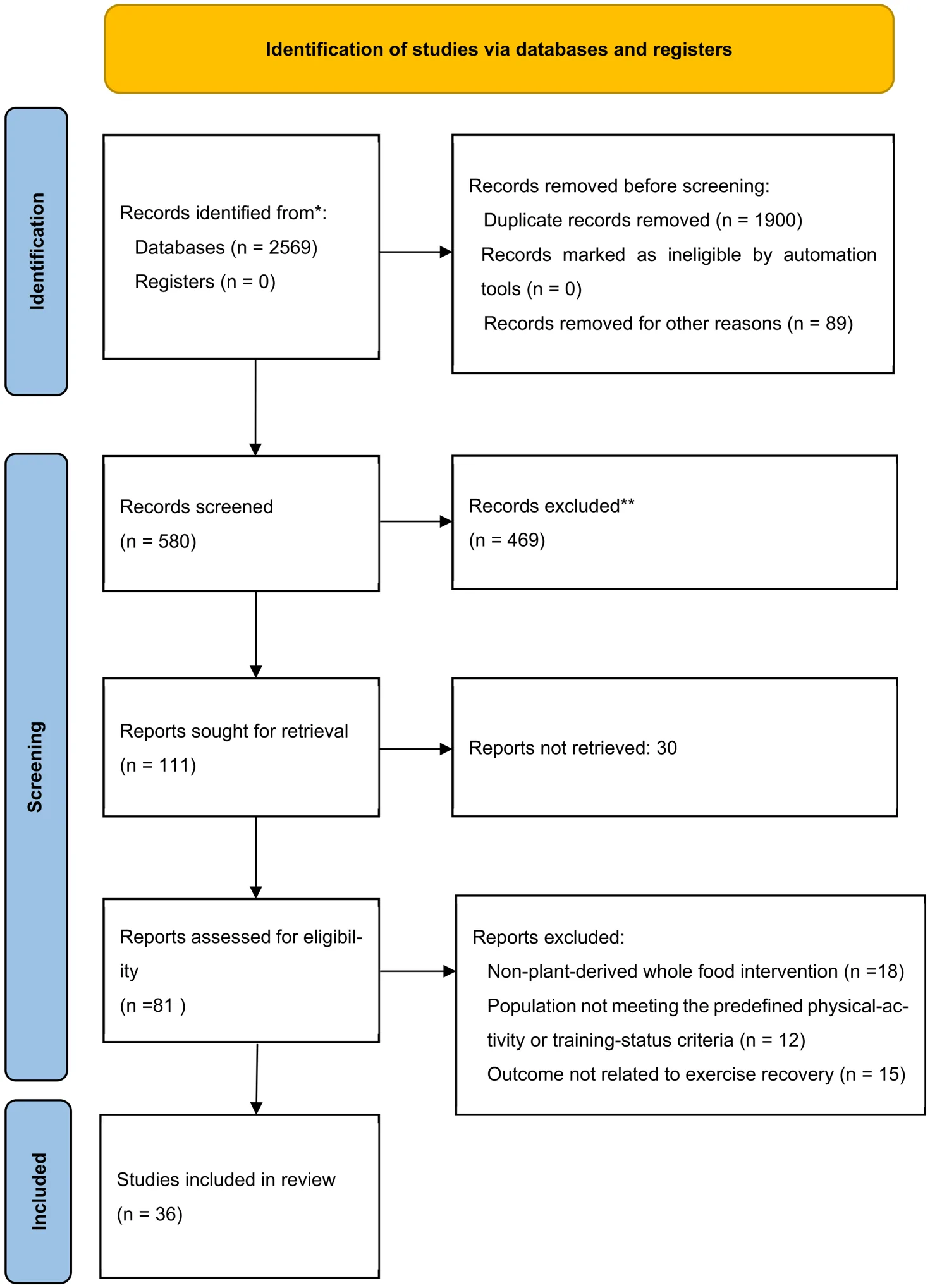
PRISMA 2020 Flow Diagram of Literature Search and Study Selection. * Records were identified from PubMed, Scopus, Embase, and Web of Science. ** Records were excluded during title and abstract screening based on the predefined eligibility criteria.

The updated database searches identified 2569 records. Before screening, 1900 duplicate records and 89 records removed for other reasons were excluded, leaving 580 records for title and abstract screening. Of these, 469 records were excluded. A total of 111 reports were sought for retrieval, of which 30 could not be retrieved. The remaining 81 full-text reports were assessed for eligibility. Forty-five reports were excluded because they evaluated an ineligible plant-derived intervention (*n* = 18), included populations that did not meet the predefined physical-activity or training-status criteria (*n* = 12), or did not report an outcome related to post-exercise recovery (*n* = 15). Finally, 36 human intervention studies were included in the systematic review (Figure 1). As summarized in Table 1, the included trials involved physically active adolescents and adults, recreationally trained participants, and competitive athletes across diverse exercise models, including eccentric exercise, resistance training, endurance events, team-sport match play, swimming, and climbing. The interventions included polyphenol-rich fruits and products such as tart cherry, pomegranate, berries, grape, and anthocyanin-rich juices; nitrate- or citrulline-rich foods such as beetroot and watermelon; carbohydrate- and electrolyte-containing foods such as banana, mango, honey, and tomato; and other plant-derived products, including Brussels chicory and avenanthramide-enriched oat products. The most frequently reported benefit was reduced muscle soreness or DOMS under selected conditions, whereas improvements in muscle function, physical performance, and biochemical markers were less consistent. Table 2 further demonstrated substantial variation in product form, bioactive composition, dose, intake frequency, intervention duration, and timing, ranging from single pre-exercise doses to supplementation protocols lasting up to 8 weeks. Dietary control also varied considerably, and adverse events were generally not reported, although one beetroot-juice study documented gastrointestinal symptoms leading to withdrawal. Supplementary Table S2 provides complementary cell-based and animal evidence indicating that plant-derived bioactives may support recovery through antioxidant and anti-inflammatory effects, preservation of mitochondrial function, stimulation of muscle protein synthesis and myogenesis, and attenuation of proteolysis and exercise-related muscle damage; however, these mechanistic findings were not included in the PRISMA efficacy synthesis and should not be interpreted as direct evidence of effectiveness in humans.

### 3.2. Study and Participant Characteristics

The 36 included studies comprised randomized controlled trials, randomized crossover trials, and controlled clinical trials conducted across the United Kingdom, the United States, Iran, Tunisia, Germany, Brazil, China, Australia, Thailand, Canada, Indonesia, Spain, and Portugal. Participants were physically active individuals aged ≥ 14 years, including adolescents and adults who were recreationally active, trained, or competitive athletes. The study populations included runners, cyclists, soccer and rugby players, volleyball players, swimmers, water-polo athletes, weightlifters, skiers, and mountain climbers. Sample sizes across the included studies ranged from 9 to 59 participants, with most trials involving relatively small study populations. Participants were predominantly young men, although some studies included women, adolescents, or mixed-sex samples. Training status varied from recreationally active individuals to highly trained and competitive athletes.

### 3.3. Intervention Characteristics

Interventions were grouped into polyphenol-rich fruit products, nitrate- or citrulline-rich foods, carbohydrate- and electrolyte-containing foods or beverages, and other plant-derived food-based products. Polyphenol-rich interventions included tart cherry, pomegranate, blueberry, bilberry, haskap berry, red berry, purple grape, and anthocyanin-rich mixed-fruit products. Nitrate- or citrulline-rich interventions mainly comprised beetroot and watermelon products. Other interventions included mango puree, Brussels chicory juice, oat avenanthramide-enriched cookies, and mixed fruit- or vegetable-based beverages. Product forms included fresh juice, concentrate, puree, freeze-dried powder, capsules, enriched cookies, and reconstituted beverages. Supplementation ranged from a single pre-exercise dose to repeated intake for up to 8 weeks, with intake occurring before exercise, during exercise, after exercise, or across the entire recovery period.

Dietary control varied considerably across studies. Some trials restricted polyphenol-, nitrate-, antioxidant-, caffeine-, alcohol-, or supplement-containing foods, whereas others relied on habitual diet records or provided limited dietary standardization. Adverse events were infrequently reported; one beetroot-juice study reported gastrointestinal symptoms leading to withdrawal, whereas most studies did not provide specific adverse-event information.

### 3.4. Recovery and Related Outcome Domains

#### 3.4.1. Muscle Soreness and Perceived Recovery

Muscle soreness was the recovery outcome for which beneficial effects were most frequently reported. Several trials involving tart cherry, pomegranate, beetroot, watermelon, and anthocyanin-rich products observed reductions in soreness or improvements in pain-related outcomes, most commonly within 24–72 h after strenuous or muscle-damaging exercise.

However, these benefits were not consistently reproduced. Several tart-cherry studies conducted in professional rugby, soccer, water-polo, and cycling populations reported no significant improvement in soreness. Beetroot and watermelon interventions also produced mixed findings, while bilberry juice was associated with transient increases in soreness and inflammatory responses following a half-marathon.

#### 3.4.2. Muscle Function Recovery

Evidence for restoration of muscle function was less consistent than that for perceived soreness. Some studies reported improved recovery of maximal voluntary contraction, countermovement-jump performance, wall-sit performance, peak torque, or lower-limb strength following tart cherry, beetroot, pomegranate, or anthocyanin-rich interventions. However, many other trials found no significant benefit for MVC or MIVC, jump height, reactive strength, range of motion, or related neuromuscular outcomes.

#### 3.4.3. Other Physical Performance Outcomes

Other physical performance outcomes reflected exercise capacity or acute performance rather than restoration of baseline muscle function. These outcomes included VO_2_ max or VO_2_ peak, time to exhaustion, running economy, time-trial performance, repeated-sprint ability, maximal power output, and sport-specific performance.

Selected benefits were reported. Haskap berry supplementation increased time to exhaustion and improved 5-km running performance, while purple grape juice prolonged time to exhaustion. Anthocyanin-rich juice accelerated the recovery of running economy after downhill running, and Brussels chicory increased exhaustion time without improving VO_2_ max. Short-term beetroot supplementation also improved repetitions to failure, movement velocity, peak power, and selected strength-related outcomes in some trials.

However, these effects were not consistently reproduced. Tart cherry juice did not improve 10-km cycling performance, repeated-sprint or water-polo performance, whereas acute beetroot juice produced no clear improvement in interval swimming performance. Across studies, both beneficial and null findings were reported for endurance-, power-, and sport-specific performance outcomes.

#### 3.4.4. Secondary Physiological Outcomes

Secondary physiological outcomes included biomarkers of muscle damage, inflammation, oxidative stress, antioxidant status, metabolic responses, cardiovascular function, and subjective recovery. Creatine kinase (CK) and lactate dehydrogenase (LDH) were the most frequently assessed markers of muscle damage. Although some studies reported lower CK or LDH concentrations at selected post-exercise time points, many found no significant between-group differences.

Inflammatory outcomes included CRP, hs-CRP, IL-6, IL-8, IL-10, TNF-α, IL-1ra, oxylipins, and immune-cell subsets, whereas oxidative-stress and antioxidant markers included MDA, protein carbonyls, lipid hydroperoxides, F_2_-isoprostanes, TAC, SOD, GPx, and glutathione-related measures. Selected interventions reduced IL-6, CRP, pro-inflammatory oxylipins, or oxidative-stress markers, or increased antioxidant capacity and skeletal-muscle antioxidant expression. Reductions in these markers or increases in antioxidant-related measures were reported in selected studies and at selected assessment time points, whereas other studies reported no significant between-group differences.

Metabolic and cardiovascular outcomes included blood lactate, glucose, heart rate, heart-rate recovery, heart-rate variability, blood pressure, muscle oxygenation, and serum potassium. Several studies reported lower lactate, heart rate, or blood pressure, as well as faster pulse recovery, whereas other studies reported no significant differences in these outcomes. Subjective recovery measures, including sleep, mood, fatigue, readiness to train, and total quality of recovery, were assessed in relatively few studies and generally showed limited or variable responses.

### 3.5. Risk of Bias (RoB 2) Summary (Qualitative)

**Figure 2:**
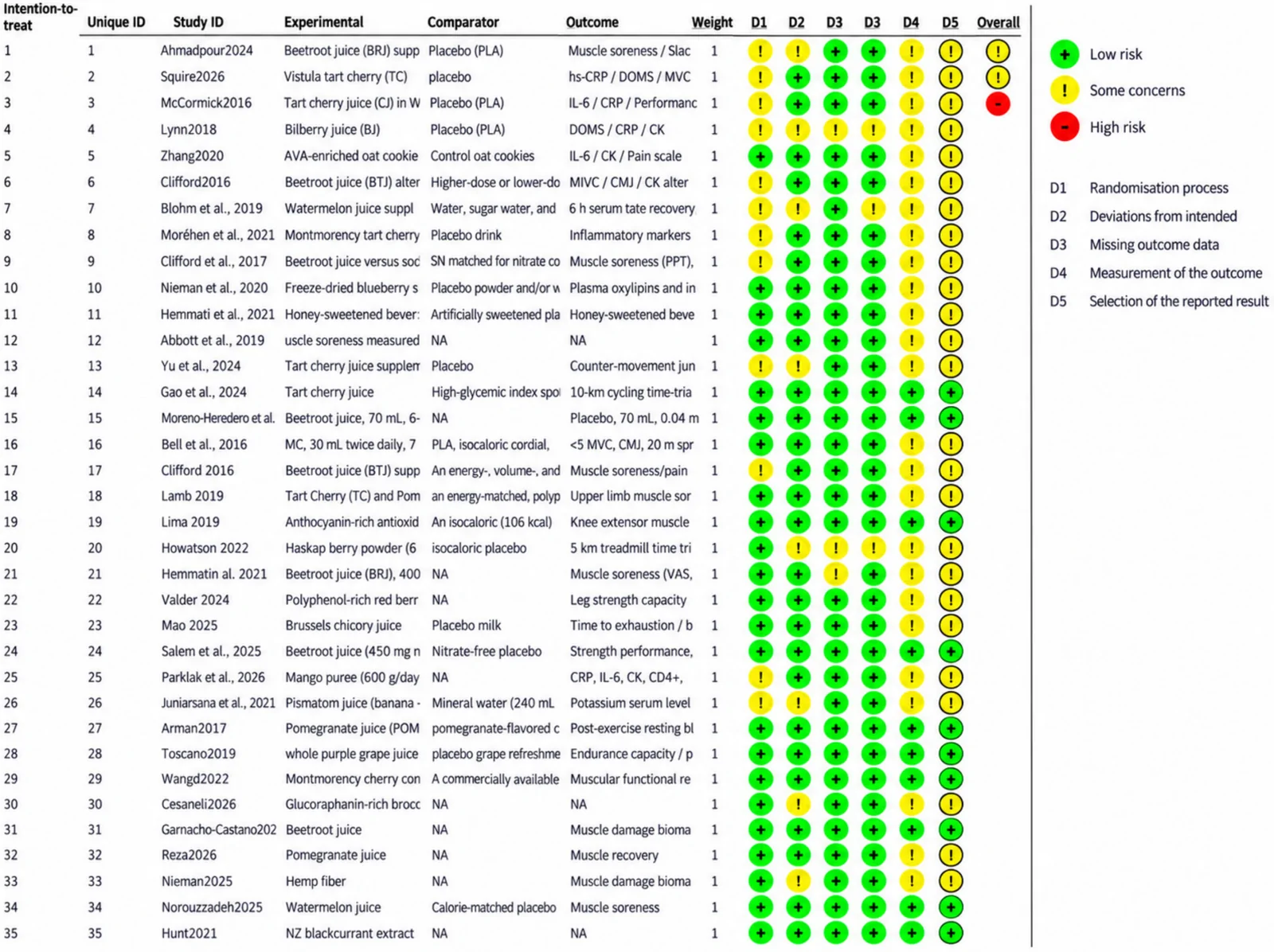
Study-level risk-of-bias judgments for the randomized human intervention trials assessed using the Cochrane RoB 2 tool (n = 35) [19,20,21,22,23,24,25,26,27,28,29,30,31,32,33,34,35,36,37,38,39,40,41,42,43,44,45,46,47,48,49,50,51,52,53]. The figure presents risk-of-bias judgments across five domains: D1, randomization process; D2, deviations from intended interventions; D3, missing outcome data; D4, measurement of the outcome; and D5, selection of the reported result. Green indicates low risk, yellow indicates some concerns, and red indicates high risk. The non-randomized study by Ammar et al. (2016) [18], which was assessed separately using ROBINS-I, is not included in this figure.

**Figure 3:**
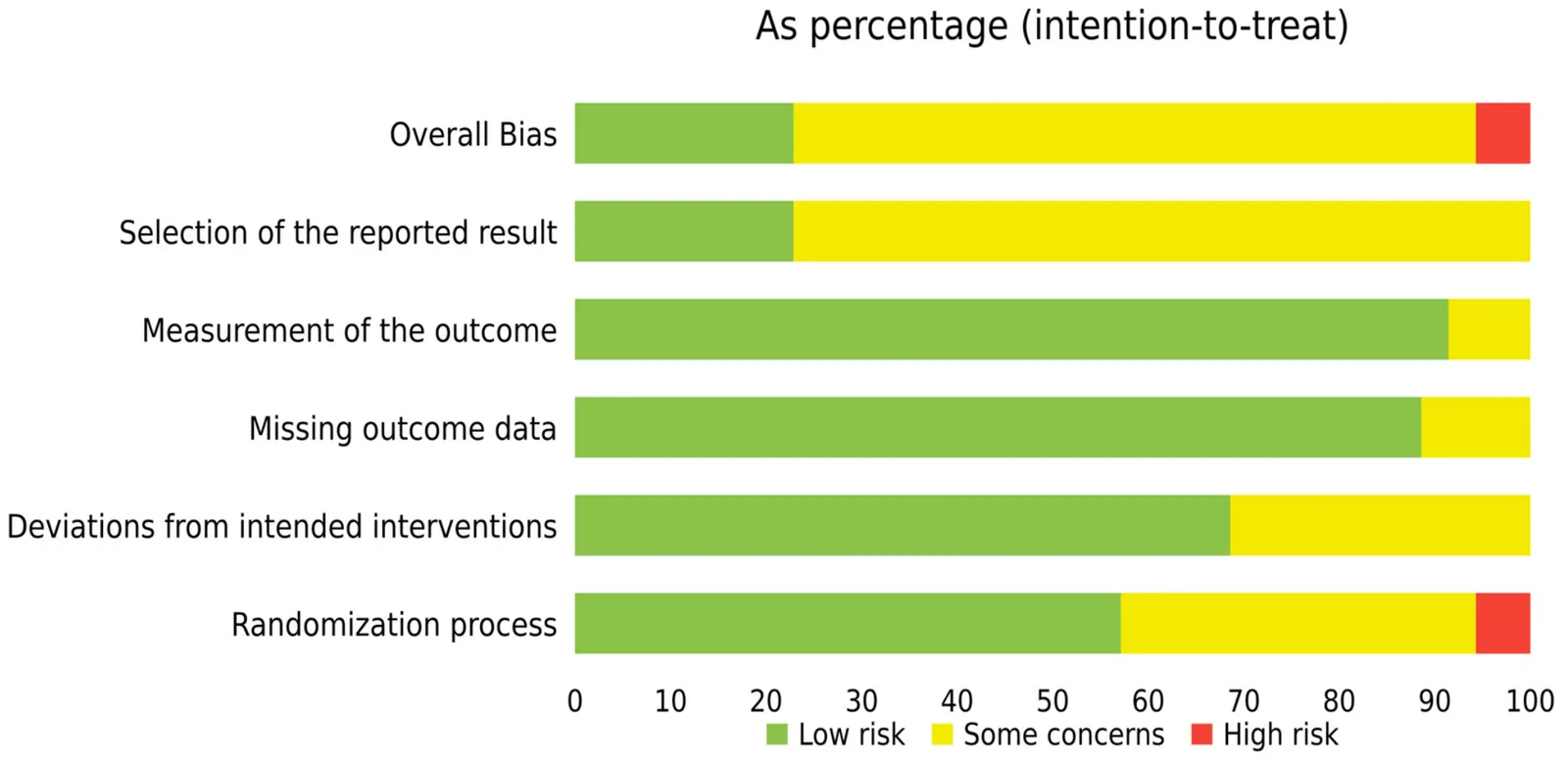
Percentage distribution of risk-of-bias judgments across the RoB 2 domains and overall assessment among the 35 randomized trials. The stacked bar chart shows the proportion of included studies rated as low risk, some concerns, or high risk for each Cochrane RoB 2 domain and for the overall assessment.

Of the 36 included human intervention studies, 35 randomized trials were evaluated using the Cochrane RoB 2 tool, whereas the non-randomized intervention study by Ammar et al. (2016) [18] was assessed separately using ROBINS-I. Among the randomized trials, 11 of 35 studies (31.4%) were judged to have a low overall risk of bias, while 24 studies (68.6%) raised some concerns. No randomized trial was judged to have a high overall risk of bias. Methodological concerns occurred most frequently in the randomization process and selection of the reported result. Because RoB 2 and ROBINS-I use different domains and judgment categories, their results were reported separately. The study-level RoB 2 judgments for the 35 randomized trials are presented in Figure 2, while the percentage distribution of domain-level and overall judgments is shown in Figure 3.

The ROBINS-I assessment of Ammar et al. (2016) [18] resulted in an overall serious risk of bias. The principal concern was bias due to confounding associated with the fixed intervention order, because all participants received the placebo condition before the pomegranate-juice condition. This design may have introduced order or repeated-bout effects that influenced the observed recovery outcomes.

### 3.6. Integrated Synthesis of Interventions, and Recovery Outcomes

Across the included studies, representative interventions included tart cherry, beetroot, pomegranate, berries, watermelon, and banana. Reductions in perceived muscle soreness and DOMS were reported in several studies, particularly following tart cherry and selected polyphenol-rich interventions, whereas other studies reported no significant effects. Both beneficial and null findings were reported for restoration of muscle function, including MVC, MIVC, CMJ, strength, and power-related performance. Biochemical outcomes, including CK, CRP, inflammatory cytokines, and oxidative-stress indices, likewise varied across studies and assessment time points.

## 4. Discussion

### 4.1. Principal Findings and Overall Interpretation

This systematic review synthesized evidence from 36 human intervention trials evaluating plant-derived whole foods and food-based supplements for post-exercise recovery. Across the narratively synthesized studies, reductions in perceived muscle soreness or delayed-onset muscle soreness were reported relatively frequently under selected exercise and supplementation conditions. However, these findings were not uniform across studies and should be interpreted cautiously given the substantial clinical and methodological heterogeneity and the absence of quantitative synthesis. In contrast, evidence for improvements in muscle-function recovery, subsequent exercise performance, and biochemical or physiological responses remained inconsistent.

The heterogeneity of the findings likely reflects substantial differences in plant source, product form, bioactive composition, dose, supplementation duration, timing of intake, participant training status, exercise protocol, dietary control, comparator condition, and outcome assessment. Training status may be particularly important because chronic exercise training induces physiological adaptations in endogenous antioxidant defenses, redox regulation, mitochondrial function, vascular function, and neuromuscular resilience. Highly trained or elite athletes may therefore exhibit smaller oxidative, inflammatory, and muscle-damage responses to a standardized exercise stimulus, potentially leaving less scope for additional benefit from polyphenol-rich or antioxidant-containing interventions. Conversely, less-trained or untrained individuals may experience greater exercise-induced physiological disturbance and may therefore show a larger apparent response to the same intervention. However, this relationship is unlikely to be linear, as periods of high training load or inadequate recovery may increase oxidative and inflammatory stress even in highly trained athletes. Accordingly, the effects of plant-derived interventions may depend partly on baseline training status, endogenous antioxidant capacity, and the magnitude of the exercise-induced disturbance. Preclinical evidence supports plausible antioxidant, anti-inflammatory, mitochondrial, anabolic, and anti-proteolytic mechanisms involving pathways such as Nrf2, NF-κB, AMPK–SIRT1–PGC-1α, and PI3K/Akt/mTOR. However, these mechanistic findings do not establish clinical effectiveness or confirm improvements in functional recovery in humans.

Overall, plant-derived interventions may support selected aspects of post-exercise recovery, particularly perceived soreness, but their effects appear to be context- and outcome-dependent. They should therefore not be interpreted as consistently improving neuromuscular function, subsequent performance, or circulating biomarkers. The following sections examine these findings separately across muscle soreness and muscle-function recovery, subsequent exercise performance, physiological and biochemical responses, and food-first implications.

**Figure 4:**
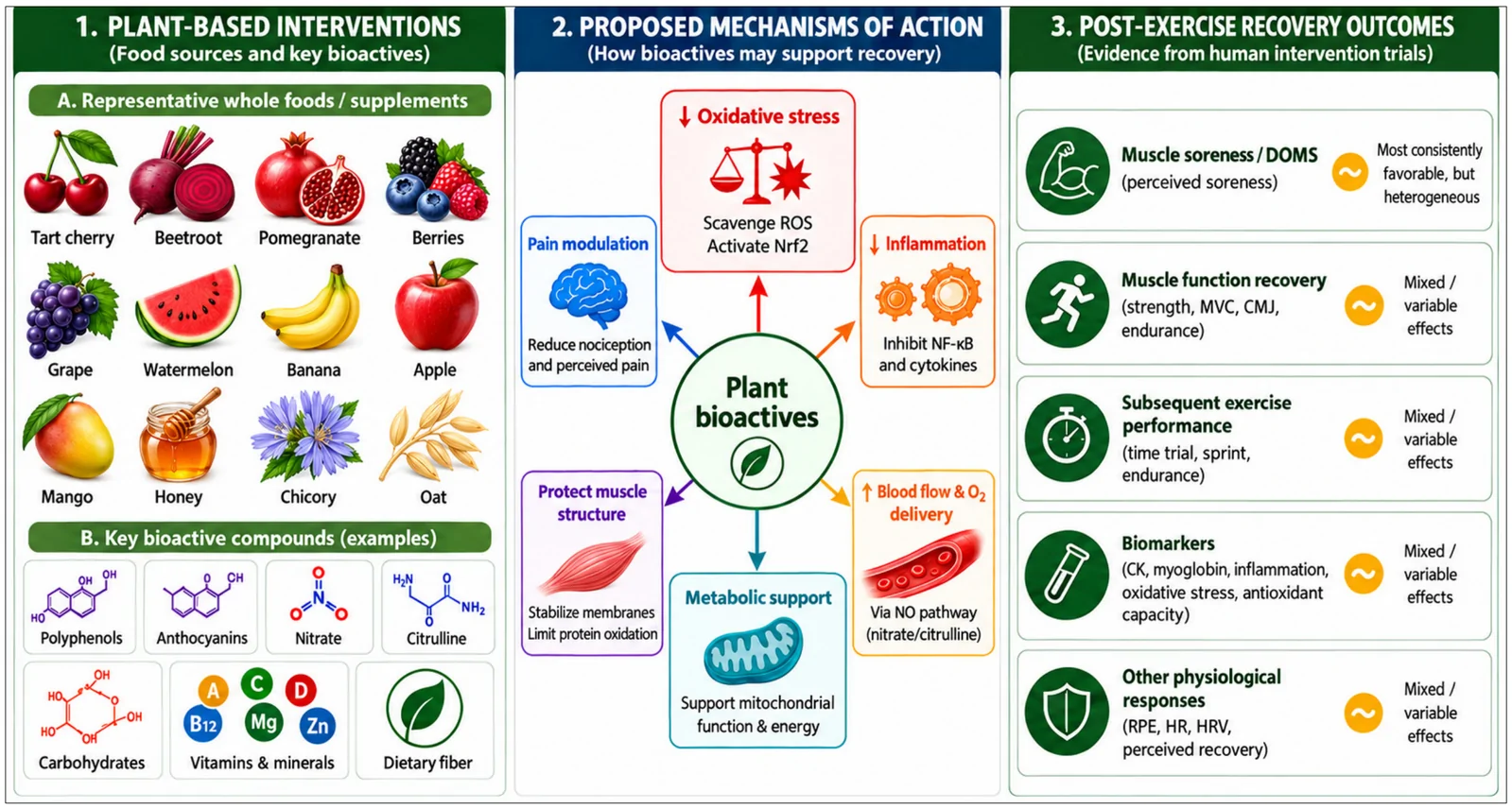
Integrated Overview of Plant-Derived Interventions, Proposed Mechanisms, and Post-Exercise Recovery Outcomes. Arrows indicate the proposed mechanistic pathways through which plant-derived bioactives may influence post-exercise recovery.

To integrate these observed outcome patterns with mechanistic interpretation, Figure 4 summarizes the principal plant-derived interventions, their major bioactive constituents, proposed mechanisms of action, and the pattern of post-exercise recovery outcomes identified in this review.

### 4.2. Muscle Soreness and Muscle Function Recovery

Reductions in muscle soreness were reported relatively frequently, although the findings were not uniform across studies. Beneficial effects were frequently observed for delayed-onset muscle soreness (DOMS), visual analogue scale (VAS) pain scores, and pressure-pain threshold, whereas improvements in objective measures of muscle function, including maximal voluntary contraction (MVC), maximal isometric voluntary contraction (MIVC), countermovement jump (CMJ) performance, peak torque, wall-sit performance, and range of motion, were less consistent across interventions, participant populations, and exercise models. This pattern is broadly consistent with previous systematic reviews reporting that plant-derived nutritional interventions more consistently attenuate perceived muscle soreness than improve objective indicators of muscle recovery [54].

The variability in findings may partly reflect differences in exercise-induced muscle damage, participant training status, intervention dose and duration, and assessment methods. VAS and Likert-type scales measure perceived discomfort, whereas pressure-pain threshold assesses mechanical pain sensitivity; although related, these outcomes are not equivalent. Complete blinding may also be difficult when interventions have distinctive colors or flavors, potentially increasing expectation-related bias. Overall, the evidence suggests that plant-derived interventions may reduce perceived soreness under selected conditions, but does not support a consistent analgesic effect across all products, populations, and exercise protocols.

Variability in muscle-function findings may also reflect differences in test reliability, participant familiarization, the muscle groups assessed, and the extent of functional impairment induced by the exercise protocol. Controlled eccentric- or resistance-exercise models may be more sensitive to detecting intervention effects than real-world match-play; however, this interpretation remains exploratory.

Evidence from a randomized controlled trial showed that a standardized lemon verbena leaf extract reduced post-exercise muscle pain and levels of creatine kinase (CK), interleukin-6 (IL-6), and urinary 8-hydroxy-2′-deoxyguanosine (8-OHdG), while increasing glutathione peroxidase (GPx) activity. However, improvements in countermovement-jump performance and isometric strength recovery were limited. Although this intervention was not included in the formal synthesis because it involved a standardized ethanol-extracted botanical product, its findings further indicate that improvements in perceived soreness and biochemical responses do not necessarily correspond to the restoration of muscle function [55].

The divergence between subjective and objective recovery outcomes likely reflects the different physiological processes underlying these measures. Perceived muscle soreness is primarily influenced by inflammation, oxidative stress, tissue edema, and nociceptor sensitization, whereas restoration of muscle function depends on recovery of excitation–contraction coupling, calcium homeostasis, contractile protein integrity, and neural activation [56,57]. Plant-derived bioactives, including anthocyanins, flavonoids, curcumin, resveratrol, quercetin, and hesperetin, may enhance antioxidant defenses, suppress inflammatory signaling, preserve mitochondrial function, and support muscle regeneration [58,59,60,61]. These mechanisms may effectively attenuate secondary muscle damage and pain perception; however, restoration of neuromuscular performance requires additional structural and functional adaptations that may occur over a longer time course, contributing to the less consistent improvements observed in muscle strength and functional performance [62,63]. Therefore, reductions in perceived muscle soreness should not be interpreted as evidence of complete physiological recovery. Future studies should assess subjective outcomes alongside objective measures of muscle function to provide a more comprehensive evaluation of post-exercise recovery.

### 4.3. Exercise Performance and Physiological Responses

Exercise performance and physiological responses were more heterogeneous than perceived muscle soreness. Some plant-derived interventions improved endurance-related outcomes, including time to exhaustion and running economy; however, effects on maximal aerobic capacity, time-trial performance, sprint ability, agility, and sport-specific performance were inconsistent. Similarly, changes in CK, LDH, CRP, inflammatory cytokines, and oxidative-stress markers varied substantially across studies. Selected improvements were observed in blood lactate clearance, heart-rate recovery, blood pressure, and other cardiovascular responses, but these effects were not consistently accompanied by enhanced exercise performance.

This variability likely reflects the multifactorial nature of physical performance, which depends on the coordinated recovery of energy metabolism, cardiovascular function, neuromuscular capacity, psychological readiness, and exercise-specific adaptations [64,65]. Consequently, improvement in a single biomarker or physiological pathway may be insufficient to produce a measurable performance benefit.

Preclinical evidence suggests that plant-derived bioactives may support recovery by preserving mitochondrial function, promoting ATP production and glycogen restoration, improving vascular function, and attenuating oxidative and inflammatory stress [58,60,66,67]. However, translation of these mechanisms into performance gains is influenced by training status, exercise modality, intervention composition, dose, and timing. Overall, plant-derived whole foods and food-based supplements may support selected physiological aspects of recovery more consistently than subsequent exercise performance. Future studies should clearly distinguish recovery outcomes from acute ergogenic effects and use standardized exercise protocols with concurrent assessment of performance, neuromuscular function, and physiological markers.

### 4.4. Effects on Subsequent Exercise Performance

Subsequent exercise performance is a practically relevant recovery outcome because it reflects the ability to complete repeated training sessions or competitive events after an initial exercise bout [68,69]. Across the included studies, however, plant-derived whole foods and food-based supplements produced inconsistent effects on subsequent performance. Benefits were reported for selected strength-, sprint-, agility-, or endurance-related outcomes, but were not consistently reproduced across exercise modalities, populations, or supplementation protocols.

Preclinical evidence suggests that plant-derived bioactives may enhance antioxidant defenses, mitochondrial function, substrate availability, muscle regeneration, and nitric oxide-mediated vascular function. However, these mechanisms represent only part of the recovery process and may be influenced by exercise type, intervention dose and timing, individual responsiveness, and the food matrix. Overall, plant-derived interventions may support selected physiological recovery processes, but current evidence does not demonstrate consistent improvements in subsequent exercise performance.

### 4.5. Food-First Implications and Quality of Evidence

The findings are compatible with a food-first approach in sports nutrition, as whole or minimally processed plant foods such as tart cherry, pomegranate, beetroot, and berries provide complementary combinations of polyphenols, anthocyanins, dietary nitrate, carbohydrates, and micronutrients within an intact food matrix [1,12]. These foods can be readily incorporated into habitual diets and may offer practical recovery options, particularly for athletes following vegetarian or vegan dietary patterns [11].

However, confidence in the evidence remains limited. Among the 35 randomized trials assessed using RoB 2, 31.4% were judged to have a low overall risk of bias, whereas 68.6% raised some concerns. In addition, the single non-randomized study was judged to have a serious overall risk of bias using ROBINS-I. These methodological limitations, together with generally small sample sizes, reduce confidence in the overall findings. Accordingly, given the substantial clinical and methodological heterogeneity across intervention–outcome comparisons, we did not assign formal outcome-specific certainty ratings, and the findings should be interpreted as a narrative synthesis rather than as graded estimates of certainty. Small sample sizes, inconsistent dietary control, and difficulties in matching the taste, color, and appearance of food-based placebos may also have reduced statistical precision and increased the potential for expectation effects, particularly for subjective soreness outcomes.

Overall, plant-derived whole foods and food-based supplements may serve as useful adjuncts for post-exercise recovery, especially for reducing perceived muscle soreness. Nevertheless, they should complement rather than replace established recovery strategies, including adequate energy and protein intake, hydration, sleep, and appropriate training management, until stronger evidence from well-designed randomized controlled trials becomes available.

### 4.6. Strengths, Limitations and Future Research Directions

This review was conducted according to PRISMA 2020 guidelines and prospectively registered in PROSPERO. It evaluated a broad range of subjective, functional, biochemical, and physiological recovery outcomes, while presenting preclinical mechanistic evidence separately from human efficacy findings to avoid overstating clinical implications.

Several limitations should be considered. Substantial heterogeneity in participant training status, exercise protocols, interventions, doses, timing, and outcome measures limited direct comparisons and quantitative synthesis. Importantly, combining elite or highly trained athletes with recreationally trained or untrained individuals may have obscured training-status-specific responses. Chronic training induces adaptations in endogenous antioxidant defenses, redox regulation, mitochondrial function, inflammatory responses, and resistance to exercise-induced muscle damage; consequently, the same plant-derived intervention and dose may not produce comparable effects across different training levels. Most studies had small samples (9–59 participants), predominantly involving young men, which reduced statistical precision and generalizability. Methodological concerns, variable dietary control, and difficulties in blinding food-based interventions further limited confidence in the findings. The restriction to English-language studies published between 2016 and 2026 may have excluded relevant evidence, while the original reliance on predefined plant-food search terms may have reduced search sensitivity. Narrative synthesis may also be influenced by reliance on statistical significance, particularly in small studies, and some studies made it difficult to distinguish acute ergogenic effects from genuine post-exercise recovery. In addition, a proportion of reports identified during screening could not be retrieved for full-text assessment. Although retrieval was attempted through the available sources, potentially eligible studies among these reports cannot be excluded, and their non-retrieval may therefore have reduced the completeness of the evidence base. Finally, the preclinical evidence was selectively identified and not formally assessed for risk of bias, and inconsistent adverse-event reporting limited conclusions regarding safety.

Future studies should use larger, adequately powered randomized controlled designs and standardized outcome sets covering muscle soreness, muscle function, performance, and biochemical recovery [70]. Future research should also apply clearly defined and standardized training-status criteria and, where feasible, investigate elite or highly trained athletes separately from recreationally trained or untrained individuals. Alternatively, training status should be prespecified as a stratification or subgroup factor to determine whether training adaptations modify responses to plant-derived recovery interventions. Follow-up should extend to 72–96 h to capture delayed responses. Improved placebo matching, standardized dietary control, prospective registration, and prespecified outcome reporting would strengthen internal validity [71,72]. Direct comparisons between whole foods and isolated phytochemicals are also needed to determine whether the food matrix provides additional recovery benefits.

## 5. Conclusions

Overall, plant-derived whole foods and food-based supplements may reduce perceived muscle soreness in some exercise and supplementation contexts; however, the findings were not consistent across interventions or studies. Evidence for restoration of muscle function, subsequent physical performance, and biochemical markers of muscle damage, inflammation, and oxidative stress remains inconclusive. Because of substantial clinical and methodological heterogeneity and the predominance of studies with some risk-of-bias concerns, no specific plant-derived intervention, dose, or timing strategy can currently be recommended based on the currently available evidence.

## Figures and Tables

**Table 1:** Characteristics and Main Findings of Human Intervention Studies on Plant-Derived Whole Foods and Food-Based Supplements for Exercise Recovery.

Author	Study Design	Participants	Exercise Protocol	Recovery Outcomes	Other Physiological/Performance Outcomes	Main Findings
Norouzzadeh et al. [19] (Iran)	Open-label RCT	42 Healthy non-athlete men (age 18–35 y)	Endurance training, 3 days/week for 8 weeks	Muscle soreness: VAS immediately and 24 h post-exercise. Muscle function recovery: 1RM strength and repetitions at 70% 1RM.	Muscle hypertrophy of pectoralis major and rectus femoris assessed by ultrasound; dietary intake; physical activity.	↓ muscle soreness at 24 h; no significant between-group differences in exercise strength or endurance.
Ahmadpour et al. [20] (Iran)	Open-label RCT	10 Expert male Alpine skiers (age 22 ± 3.6 y)	90-s box jump, hexagonal jump, wall-sit, and two altitude slalom runs; performance and muscle soreness measured	Muscle soreness: VAS at baseline, 12, 24, and 48 h post-exercise. Muscle function recovery: wall-sit, 90-s box jump, hexagonal jump, slalom time	RPE immediately after slalom runs.	↓ muscle soreness at 12–48 h; ↑ wall-sit, 90-s box-jump, and agility performance; ↓ RPE; no significant effect on slalom time
Hemmatinafar et al. [21] (Iran)	Randomized, crossover, placebo-controlled, double-blind trial	12 Semi-professional female volleyball players; (age 26.0 ± 3.0 y)	Exercise-induced muscle damage protocol: 200 vertical jumps with 10% body-weighted vest; 10 sets of 20 maximal jumps; 2 min rest between sets	Muscle soreness: VAS muscle soreness at 12, 24, and 48 h after EIMD. Muscle function recovery: wall-sit, vertical jump height, V-sit and reach flexibility test, and pressure pain threshold at 48 h after EIMD	Thigh swelling. No CK, LDH, CRP, IL-6, TNF-α, oxidative stress, or metabolic biomarkers measured	↓ soreness/swelling and improved wall-sit; no effect on VJH, VSFT or PPT
Hemmati et al. [22] (Iran)	Randomized, crossover, placebo-controlled, double-blind trial	16 Strength-trained females (age 27 ± 4 y)	EIMD induced by 200 weighted vertical jumps (10 × 20, 10% body weight); outcomes assessed 48 h later	Muscle soreness: VAS from baseline to 48 h post-EIMD. Muscle function recovery: wall-sit, 1RM leg press, vertical jump, V-sit and reach, and pressure pain threshold	RPE immediately after EIMD. No CK, LDH, inflammatory, oxidative stress, or metabolic biomarkers measured	↓ DOMS/RPE and ↑ wall-sit and 1RM; no effect on VJH, PPT or flexibility
Nieman et al. [23] (USA)	Randomized, double-blind, placebo-controlled, parallel four-group trial	59 Trained male and female cyclists (age 18–55 y)	75-km cycling time trial lasting approximately 185.5 ± 5.2 min, followed by 48 h recovery monitoring.	Not assessed	Plasma oxylipins, ARA/DHA/EPA-derived oxylipins, gut-derived phenolics, serum glucose, IL-6, IL-1ra	↓ pro-inflammatory ARA-CYP oxylipins during early recovery.
Valder et al. [24] (Germany)	Open-label RCT	18 Recreational endurance athletes 14 men and 4 women (age 24.5 ± 3.1)	Six-day intensive endurance protocol: four HIIT sessions and two endurance runs. Interval sessions were performed at 120% of individual lactate threshold; endurance runs at 65% of lactate threshold.	Muscle soreness: not assessed Muscle function recovery: estimated 1RM and relative estimated 1RM in deep back squat before and after the 6-day endurance protocol.	CK. Oxidative stress: oxLDL, IL-6 and IL-10.	No significant effect on muscle damage, oxidative stress, inflammation or leg strength.
Howatson et al. [25] (UK)	Randomized, double-blind, placebo-controlled	30 Male recreational runners (age 18–45 y)	Submaximal lactate profile, VO_2_ peak test to exhaustion, and 5 km treadmill time trial	Muscle soreness: not assessed Muscle function recovery: Not assessed as recovery; performance tests only	VO_2_ peak test time to exhaustion, 5 km time-trial time and mean speed, heart rate, VO_2_, RPE, lactate	Haskap ↑ exhaustion time by 20 s and improved 5-km time by 21 s.
Gao et al. [26] (Canada)	Randomized, double-blind, placebo-controlled	12 Recreational cyclists; 8 males and 4 females (age 35 ± 16 y)	90 min cycling at approximately 65% VO_2_ peak	Muscle soreness: VAS-based pain score after pressure application to knee extensors. Muscle function recovery: knee-extensor MVC and low-frequency fatigue (P20/P80) at baseline, post 24 h, and 48 h	10 km time trial, blood glucose, lactate, carbohydrate oxidation, fat oxidation, RER, VO_2_/O_2_ cost, RPE, and 24-h blood pressure	Not improve performance, metabolism or recovery
Yu et al. [27] (China)	Randomized, double-blind, placebo-controlled	24 Male college soccer players (age 22 ± 3)	Periodized resisted sled-based training in week 4 after 3 weeks of supplementation.	Muscle soreness: CMJ, knee flexion strength Muscle function recovery: Nordic hamstring performance	Seated/prone knee-flexion strength and Nordic hamstring performance	TC attenuated strength loss and accelerated recovery up to 72 h.
McCormick et al. [28] (Australia)	Randomized, double-blind	9 Highly trained male water polo athletes (age 18.6 ± 1.4 y)	Seven-day water polo training protocol.	Muscle soreness/DOMS: daily DOMS ratings for upper body, upper legs, lower legs, and overall body. Function/performance-related recovery: water-based vertical jump, 10 m sprint, repeated sprint test, and Water Polo Intermittent Shuttle Test	IL-6, CRP, uric acid, F2-isoprostane, lactate, total quality of recovery, training load	Not improve performance, soreness or biochemical recovery.
Clifford et al. [29] (UK)	Double-blind, placebo-controlled	30 Recreationally active men (age 22.6 ± 2.8 y)	100 drop jumps from a 0.6-m box; 10-s intervals and 2-min rest after every 20 jumps	Muscle function recovery: MIVC, CMJ and PPT	CK, hs-CRP and serum NOx	Reduced muscle pain but did not improve muscle function or biochemical recovery
Clifford et al. [30] (UK)	Double-blind, placebo-controlled	20 Male collegiate team-sports players (age 23 ± 3 y)	Two repeated sprint tests separated by 72 h	Muscle soreness/pain: pressure-pain threshold Muscle function recovery: CMJ, reactive strength index, MIVC.	Sprint performance, RPE, CK, hs-CRP, protein carbonyls, lipid hydroperoxides, ascorbic free radical	BTJ improved CMJ and RI recovery; no effect on sprint performance, MIVC or biomarkers.
Squires et al. [31] (UK)	Randomized, double-blind, placebo-controlled, parallel-group repeated-measures trial	Recreationally trained marathon runners; 23 males; 12 females;	Undulating marathon race; assessments before, immediately after, 24 h, and 48 h post-marathon	Muscle soreness/DOMS: active lower-limb soreness using VAS; passive soreness. Muscle function recovery: MVC and CMJ assessed before, immediately after, 24 h, and 48 h post-marathon	CK, hs-CRP, fatigue, energy, sleepiness, readiness to train.	Reduced DOMS; improved MVIC; no effect on hs-CRP.
Blohm et al. [32] (UK)	Randomized crossover trial	27 Healthy non-athletic; 13 males and 14 females (age 21–35 y)	Submaximal cycling test until 80% predicted HRmax; post-exercise monitoring up to 20 min; soreness assessed 24 h post-exercise	Muscle soreness/DOMS: 24-h post-exercise muscle soreness scale. Muscle function recovery: Not assessed	Heart rate recovery, blood lactate, blood pressure, blood glucose	WJ had no effect on HR recovery, lactate, soreness or performance; prevented post-exercise BP rise in females
Clifford et al. (UK) [33]	Randomized, double-blind, placebo-controlled, independent-groups trial	29 Recreationally active healthy males; (age 21 ± 3 y)	Two bouts of eccentric exercise: 100 drop jumps, separated by 14–21 days; outcomes measured pre, post, 24, 48, and 72 h	Muscle soreness/pain: pressure-pain threshold. Muscle function recovery: CMJ, reactive strength index, MIVC	CK; repeated-bout effect as adaptation outcome	not impair repeated-bout adaptation, regardless of dose
Zhang et al. [34] (USA)	Double-blind, randomized controlled study	Healthy, non-obese, insufficiently active men and women	Downhill running at −10% grade, 4 × 15 min at 75% HRmax	Muscle soreness/DOMS: pain rating scale after squat up to 72 h after downhill running. Muscle function recovery: not assessed	CK, neutrophil respiratory burst, G-CSF, IL-6, IL-1Ra, sVCAM-1, MCP-1	AVA ↓ NRB, G-CSF and sVCAM-1; ↑ IL-1Ra; no clear AVA-specific effect on CK or soreness.
Lynn et al. [35] (UK)	Single-blind, randomized, placebo-controlled, parallel-group trial	21 Recreationally trained half-marathon runners; 16 males, 5 females (age 30.9 ± 10.4 y)	Sheffield Half Marathon	Muscle soreness: lower-body DOMS using 200-mm VAS after squat. Muscle function recovery: Not assessed	CK, CRP	Increased DOMS and CRP; no effect on CK
Ammar et al. [18] (Tunisia)	Non-randomized, placebo-controlled trial	9 Elite male weightlifts (age 21 ± 1 y)	Two Olympic- weightlifting sessions (snatch, clean and jerk, and squat); 5 sets per exercise at 85–90% of 1-RM; session duration: 1 h 46 min)	Muscle soreness: DOMS of knee extensors and elbow flexors 48 h post-training Muscle function recovery: Weightlifting volume and maximal load; performance-related rather than direct recovery	CK, LDH, ASAT, BP, HR, CRP, RPE, hematological markers	Reduced DOMS and muscle-damage/inflammatory markers and improved weightlifting recovery up to 48 h after exercise.
Morehen et al. [36] (UK)	Single-blind randomized cross-over design	11 Professional male rugby league players (age 18 ± 1 y)	Two professional rugby league matches	Muscle soreness: 1–5 Likert scale at 24 h pre-match and 24/48 h post-match. Muscle function recovery: CMJ and drop jump at 48 h pre/post-match.	IL-6, IL-8, IL-10, sleep, fatigue, mood, stress, subjective wellness	MC did not improve inflammation, soreness, muscle function or wellness.
Abbott et al. [37] (UK)	Randomized, double-blind, placebo-controlled, crossover design	10 Male professional soccer players (age 19 ± 1 y)	90-min competitive soccer match	Muscle soreness: using a 0–200 mm visual analogue scale Muscle function recovery: CMJ and reactive strength index	Subjective well-being: fatigue, sleep, mood, stress	Not improve muscle function, soreness or well-being.
Moreno-Heredero et al. [38] (Spain and Portugal)	Cross-over, randomized, placebo-controlled, double-blind, and counterbalanced study	Competitive swimmers (9 females and 9 males)	2 × 6 × 100 m maximal effort front crawl with 40 s rest between repetitions and 3 min rest between blocks	Muscle soreness: Not assessed Muscle function recovery: CMJ after resting values; recovery context should be verified	Blood lactate, heart rate, RPE, total quality recovery	Not improve overall performance; possible benefit for final-repetition tolerance and perceived recovery.
Lamb et al. [39] (UK)	Randomized, double-blind, placebo-controlled, parallel study	Non-resistance-trained men	High-force eccentric exercise of the elbow flexors: 5 sets of 10 maximal voluntary eccentric contractions on an isokinetic dynamometer	Muscle Soreness: Elbow flexor soreness rated on 100-mm VAS Muscle function recovery: MIVC and ROM measured up to 96 h post-exercise	ROM and CK	Neither TC nor POM improved MIVC, DOMS, ROM or CK recovery.
Lima et al. [40] (Brazil)	Double-blind, placebo -controlled, randomized	Healthy young male students	30-min downhill running	Muscle Soreness: lower-limb muscle soreness assessed before and 1–4 days after downhill running. Muscle function recovery: knee-extensor isometric peak torque.	running economy markers, VO_2_, perceived effort, serum CK.	Accelerated recovery of running economy and IPT and reduced soreness and CK
Mao et al. [41] (China)	Pilot randomized, single-blind, placebo-controlled, two-way crossover study	Healthy, untrained college students	A short-term, progressive workload, high-intensity, exhaustive aerobic exercise with the Bruce protocol	Muscle soreness: not assessed Muscle recovery: not assessed	VO_2_max, HR, BP, IL-6, IL-8, CRP, SOD, GPx, CAT, blood pH, bicarbonate	Chicory ↑ exhaustion time; ↓ lactate, HR and SBP; no effect on VO_2_ max or inflammatory markers.
Salem et al. [42] (Tunisia and Germany)	Randomized, double-blind, crossover, within-subject controlled trial	12 Healthy, physically active, resistance-trained males (age 21.3 ± 1.9 y)	Incremental back squat and bench press exercises	DOMS: using VAS 0–10 Muscle recovery: Repetition to failure, movement velocity, peak power, CMJ/squat jump if assessed during recovery	Heart rate, muscle oxygenation, HRV, blood lactate	↑ repetitions, velocity and SmO_2_; ↓ HR and DOMS; accelerated jump recovery.
Parklak et al. [43] (Thailand)	Pilot randomized, single-blind, crossover trial	15 Thai men’s national beach volleyball players (age 24 ± 3 y)	A regular national team training program	No assessed	CK, CRP, IL-6, selected T-cell subsets, resting HR, body composition, BP	Mango ↓ CRP, IL-6 and CK; ↑ CD4+ and CD4/CD8 ratio.
Juniarsana et al. [44] (Indonesia)	Randomized pre-test-post-test control group experimental design	28 Middle run athletes, male sex (age 14–18 y)	Middle-distance running	Not assessed	Serum potassium recovery; 5-min pulse recovery rate	↑ serum K^+^ and accelerated heart-rate recovery at 3–5 min.
Daneshparvar et al. [45] (Iran)	Randomized, placebo-controlled, double-blinded study	27 Experienced mountain climbers; 14 males and 13 females (age 37 ± 7 y)	Climbing Riz o Boland Mountain to a summit of 3720 m	Muscle soreness: VAS soreness of quadriceps, hamstrings, gastrocnemius; pressure pain threshold Muscle function recovery: isokinetic and isometric strength tests; aerobic power tests	thigh swelling	Improved several strength/performance measures and ↓ 24-h gastrocnemius DOMS vs. control.
Toscano et al. [46] (Brazil)	Randomized, crossover, double-blind, placebo-controlled clinical trial	14 Male recreational runners (age 39 ± 9 y)	Two running tests to exhaustion at 80% of VO2max	Not assessed	MDA, hs-CRP, A1GPA, CK, LDH, mood, stress, recovery, sleep quality, total antioxidant capacity	↑ exhaustion time by 18.7% and TAC; no effect on inflammation or muscle-damage markers.
Wangdi et al. [47] (UK)	Double-blind, crossover design	10 Recreationally active males (age 23.4 ± 5.4)	Two maximal unilateral eccentric knee extension trials	Muscle soreness/DOMS: knee-extensor soreness assessed before, immediately, 24 h, and 48 h after exercise. Muscle function recovery: knee-extension maximal voluntary contractions, maximal isokinetic contractions, single-leg jumps	Muscle mRNA/protein expression including SOD/GPX; phenolic metabolites; IL-6, TNF-alpha, CRP, CK	MCC preserved MVC and ↑ GPX gene/protein expression; no effect on soreness or blood biomarkers.
Bell et al. [48] (UK)	Randomized, double-blind, placebo-controlled, parallel-group trial	16 Male semi-professional soccer players (age 25 ± 4 y)	Adapted Loughborough Intermittent Shuttle Test: 12 × 20-m sprints followed by 6 × 15-min intermittent shuttle-running sections	Soreness: 200-mm VAS at baseline and 24, 48, and 72 h. Muscle function: knee-extensor MVIC and CMJ at the same time points	20-m sprint and 5-0-5 agility recovery. CK, IL-1β, IL-6, IL-8, TNF-α, hs-CRP, and LOOH	Reduced DOMS and accelerated recovery of MVIC, CMJ, sprint, and agility; attenuated IL-6, with no significant effects on CK or oxidative stress changed
Hunt et al. [49]	Double-blind, randomized, placebo-controlled, parallel-group trial	14 Healthy non-resistance-trained men and women (age 24 ± 2 y)	4 × 15 maximal concentric and eccentric contractions of the dominant-arm biceps brachii using an isokinetic dynamometer	Muscle soreness: VAS at 24, 48, 72, and 96 h. Muscle-function recovery: isometric MVC and elbow ROM	Serum CK and mid-arm circumference	NZBC extract accelerated MVC recovery, reduced muscle soreness at 24 and 48 h, and lowered serum CK at 96 h. No significant between-group differences were observed for ROM or mid-arm circumference.
Nieman et al. [50]	Randomized, placebo-controlled, double-blind, crossover trial	23 Healthy male and female cyclists (age M: 45.5 ± 2.2 y; F: 46.5 ± 4.1 y)	2.25-h intensive cycling bout (~70% VO_2_ max) after 2 weeks of supplementation	Muscle soreness: DOMS (1–10 scale) assessed pre-exercise, immediately post-exercise, 1.5 h and 3 h post-exercise. Muscle damage: serum CK and myoglobin assessed pre- and post-exercise	Gut permeability (urinary lactulose/^13^C-mannitol ratio); cortisol; blood cell counts; plasma untargeted metabolomics; cycling power, HR, VO_2_, distance and speed	DOMS, CK and myoglobin increased after exercise, but no significant supplement × time differences between high-dose hemp fiber, low-dose hemp fiber and placebo. Hemp fiber altered several plasma metabolites but did not significantly affect post-exercise gut permeability.
Rezaei et al. [51]	Randomized, placebo-controlled, double-blind, crossover trial	14 Tier-3 highly trained collegiate male volleyball players (age 22 ± 2 y)	EIMD protocol: 200 maximal vertical jumps wearing a vest equal to 10% body mass (10 × 20 jumps)	Muscle soreness: DOMS (VAS) at baseline, 0, 12, 24 and 48 h. Muscle function recovery: VJH, handgrip strength, medicine-ball throw, wall-squat; knee extensor/flexor isokinetic and isometric strength (APT, RPT, TPT, AvRFD, AvP, MVIC) at baseline and 48 h	Flexibility: back-scratch and V-seat-and-reach; individual responder analysis using smallest worthwhile change (SWC)	Pomegranate juice did not significantly outperform placebo for group-mean DOMS or functional/isokinetic recovery at 48 **h**. However, SWC analysis indicated a greater proportion of POMj responders for selected high-velocity isokinetic outcomes.
Garnacho-Castaño et al. [52]	Randomized, double-blind, crossover trial	11 Well-trained male CrossFit athletes (age 29.2 ± 3.7 y)	Two resistance-exercise routines consisting of 90-s wall-ball shots + 60-s full back squat (50% 1RM), performed with and without a 3-min rest between exercises	Muscle damage: serum CK and LDH, plus myoglobin, assessed pre- and immediately post-exercise	Repetitions; VO_2_; VCO_2_; ventilation; RER; HR; plasma nitrate + nitrite (NOx)	Beetroot juice increased full-back-squat repetitions after the rest period and reduced VO_2_ during rest/full-back-squat exercise. CK and LDH increased after exercise but did not differ significantly between BJ and placebo; myoglobin increased more with BJ.
Cesanelli et al. [53]	Randomized, double-blind, placebo-controlled, crossover trial	15 Healthy untrained males (age 26 ± 6 y)	Elbow-flexor EIMD: 6 sets × 10 maximal eccentric contractions at 60°/	Muscle soreness: 10-point VAS. Muscle function recovery: isometric and isokinetic peak torque. Muscle damage: plasma CK. All assessed baseline, immediately post, 48 h and 96 h	Elbow ROM; arm girth; ultrasound muscle thickness and CSA; distal tendon thickness; muscle/tendon echo intensity	Eccentric exercise caused significant strength loss, soreness, increased CK and structural changes, but no significant Time × Supplement interactions. Short-term glucoraphanin-rich broccoli powder did not improve recovery compared with placebo.

Data are presented as reported in the original studies; age is expressed as mean ± SD or range, as applicable. Primary and secondary outcomes were classified by the review authors according to the predefined outcome domains and may not correspond to the outcomes designated as primary or secondary in the original articles. ↑ indicates a significant increase or improvement; ↓ indicates a significant decrease or attenuation; NR, not reported; 1RM, one-repetition maximum; BP, blood pressure; CK, creatine kinase; CMJ, countermovement jump; CRP, C-reactive protein; DOMS, delayed-onset muscle soreness; EIMD, exercise-induced muscle damage; GPx, glutathione peroxidase; HR, heart rate; HRV, heart-rate variability; hs-CRP, high-sensitivity C-reactive protein; IL, interleukin; LDH, lactate dehydrogenase; MDA, malondialdehyde; MIVC/MVIC/MVC, maximal isometric voluntary contraction/maximal voluntary isometric contraction/maximal voluntary contraction; oxLDL, oxidized low-density lipoprotein; PPT, pressure-pain threshold; RPE, rating of perceived exertion; RSI/RI, reactive strength index; SOD, superoxide dismutase; TAC, total antioxidant capacity; TNF-α, tumor necrosis factor alpha; VAS, visual analogue scale; VO_2_ max/VO_2_ peak, maximal/peak oxygen uptake.

**Table 2:** Intervention Characteristics of Plant-Derived Whole Foods and Food-Based Supplements Included in Human Studies.

Author	Plant Source	Product Form	Main Bioactive Compounds	Dose	Frequency	Duration	Timing of Intake	Dietary Control	Adverse Events
Norouzzadeh et al. [19]	Watermelon	Fresh juice	L-citrulline, lycopene, carotenoids, glutathione, carbohydrate	710 mL; approximately 1.65 g L-citrulline	Before each exercise session	8 weeks	1 h pre-exercise	3-day 24-h dietary recalls	NR
Ahmadpour et al. [20]	Beetroot	Beetroot juice	Dietary nitrate/nitrite–NO pathway compounds	220 mL beetroot juice; nitrate reported as approximately 8.9 mmol/L	Single dose per test session	Acute; two experimental sessions	2.5 h before functional tests and slalom runs	Food diary and prohibited-food questionnaire; nitrate-rich foods restricted	NR
Hemmati et al. [22]	Honey	Honey-sweetened beverage	Fructose, glucose, sucrose, phenolic/flavonoid compounds, vitamins and minerals	70 g honey in 250 mL water; 28% concentration	Single dose per condition	Acute crossover trial; 1-week washout	90 min before EIMD	Standardized breakfast; dietary/exercise restrictions for 24 h pre-trial; testing during follicular phase	NR
Nieman et al. [23]	Blueberry and banana	Freeze-dried blueberry powder	Blueberry: polyphenols/anthocyanins; Banana: carbohydrates.	Blueberry 26 g/day; banana 0.2 g carbohydrate/kg every 15 min during cycling	Blueberry daily; banana every 15 min during exercise	Blueberry for 2 weeks; banana acute during 75-km cycling	Blueberry at first meal; final dose during recovery meal; banana during exercise	3-day controlled diet; food records; standardized recovery meal	NR
Hemmatinafar et al. [21]	Beetroot	Beetroot juice	Nitrate, betaines, flavonoids, phenolic compounds, carbohydrates	400 mL over 2 days; nitrate 4.1 mmol/L	Eight servings of 50 mL	48 h after EIMD	2, 6, 10, 14, 26, 30, 34, and 38 h after EIMD	Diet and training supervised; 100 g banana before EIMD	Two participants withdrew due to digestive disorder
Valder et al. [24]	Red berry/chokeberry mixed fruit drink	Polyphenol-rich mixed fruit juice drink	Polyphenols, phenolic acids, proanthocyanins, anthocyanins, flavanols, flavanones	400 mL/day; 200 mL morning and 200 mL evening; approximately 1.64 g polyphenols/day	Twice daily	3-day familiarization plus 6-day training intervention	Morning and evening after meals	Nutrition app; diet replicated where feasible; high-polyphenol foods restricted.	NR
Howatson et al. [25]	Haskap berry	Freeze-dried Haskap berry powder mixed with low fat yogurt	Anthocyanins, especially cyanidin-3-O-glucoside	6 g powder per dose; approximately 24.9 mg anthocyanins/g	Daily for 6 days plus acute doses on test day	6 consecutive days	Each morning; additional doses before tests	habitual diet and exercise routines but restricted high-polyphenol/anthocyanin foods to one portion/day.	NR
Gao et al. [26]	Tart cherry	Tart cherry juice	Anthocyanins, flavonoids, carbohydrates	Days 1–4, 6, and 7: 150 mL per dose	Twice daily	4 days before exercise, exercise day, and 2 days after exercise	Breakfast and bedtime; exercise-day pre-exercise dose 45 min before exercise	Polyphenol-rich foods minimized; diet/activity replicated	NR
Yu et al. [27]	Tart cherry/Montmorency cherry	Tart cherry concentrate/juice	Polyphenols, anthocyanins, hydroxycinnamates, flavanols, melatonin, serotonin, tryptophan, vitamin C, carotenoids, potassium	70 mL per serving	Twice daily	3 weeks before resisted sled training	9:00 AM and 5:00 PM daily	Dietary intake was controlled; polyphenol foods/alcohol restricted	NR
Clifford et al. [30]	Beetroot juice	Beetroot juice; 99% beetroot juice concentrate	Nitrate, betalains, phenolic compounds, antioxidant constituents	2 × 250 mL per serving; each serving provided 81 kcal	Two servings/day during recovery	From immediately after RST1 through the recovery period and after RST2	One serving 30 min after each RST and one serving with the evening meal	24 h dietary recall	NR
McCormick et al. [28]	Tart Montmorency cherry	Tart cherry juice concentrate diluted with water	Anthocyanins and phytochemicals	90 mL concentrate/day; each 30 mL serving diluted to 200 mL	Twice daily	6 consecutive days	200 mL before morning training and 400 mL in the evening after training	Fixed training schedule during both trials.	NR
Squires et al. [31]	Vistula tart cherry/Nadwiślanka cherry	Spray-dried tart cherry extract in capsule form	Anthocyanins expressed as cyanidin-3-glucoside; total polyphenols	Lower dose first 4 days: 68.4 mg total anthocyanins and 339.9 mg total polyphenols	Twice daily	7 days total; marathon on day 5	Loading phase 4 days before marathon; continued on marathon day and 2 days post-marathon	habitual diet and logged high-polyphenol foods	NR
Blohm et al. [32]	Watermelon	Watermelon juice	L-citrulline, potassium, carbohydrate; possible NO-related effects	355 mL; 780 mg L-citrulline	Single dose	Acute	45 min before submaximal cycling	Avoided watermelon/products/supplements; caffeine, alcohol, exercise restricted 24 h before testing	NR
Zhang et al. [34]	Oat	Avenanthramide-enriched oat cookies	Avenanthramides; oat polyphenols	2 cookies/day; total 20.6 mg AVA/day	Daily supplementation before second downhill running protocol	8 weeks	NR	Excluded nutraceuticals/vitamin supplements and high exercise volume; detailed dietary control	NR
Lynn et al. [35]	Bilberry	Bilberry juice	Polyphenols and anthocyanins	2 × 200 mL/day; each 200 mL contained about 744 mg total phenols and 80 mg anthocyanins	Twice daily	8 days—5 days before the half-marathon, race day, and 2 days afterward	One serving with breakfast and one with the evening meal	Food diary; NSAIDs and antioxidant supplements avoided	NR
Ammar et al. [18]	Pomegranate	Natural pomegranate juice	Polyphenolic antioxidant compounds and anthocyanins and ellagitannins	250 mL per serving during preloading; additional 500 mL test-day dose. Total: 2.0 L per condition	Three times daily during the pre-loading phase	Over the 48 h preceding the training session, plus the day of the session	Preloading at 07:00, 15:00, 23:00; test-day dose 60 min before training	Dietary records; antioxidants/anti-inflammatory drugs and polyphenol-rich foods avoided	NR
Morehen et al. [36]	Montmorency cherries	Montmorency tart cherry juice concentrate	Anthocyanins	30 mL of concentrate mixed with 100 mL of water	Twice daily	5 days before match, match day, and 2 days after	morning and evening	Habitual nutritional intake	NR
Abbott et al. [37]	Tart cherry	Tart cherry juice concentrate	Flavonoids	30 mL gel mixed with water to make 250 mL serving	Twice daily	Match morning to 36 h post-match (about 3 days)	Morning and within 30 min post-match/training	Habitual diet	NR
Moreno-Heredero et al. [38]	Beetroot	Concentrated beetroot juice shot	Nitrate	70 mL	Single dose	A single testing session	3 h pre-exercise	Standardized macronutrient intake; avoided nitrate-rich foods, mouthwash, stimulants, and alcohol.	NR
Lamb et al. [39]	Tart cherry and Pomegranate	Tart cherry: 30 mL concentrate diluted with 220 mL water. Pomegranate: 250 mL undiluted juice	Polyphenols, anthocyanins, ellagitannins, and flavan-3-ols	250 mL per serving	Twice daily	9 days	One dose in the morning and one in the evening	Avoided strenuous activity, NSAIDs, polyphenol-rich foods, and antioxidants.	NR
Lima et al. [40]	Apple, plum, blueberry, maqui berry, raspberry, cranberry	Anthocyanin-rich antioxidant juice	Anthocyanins	240 mL per dose	Twice daily	4 days before downhill run, day of run, and 4 days after	Doses were taken with a 12-h interval	Regular diet maintained; intense activity avoided; hydration encouraged	NR
Mao et al. [41]	Brussels chicory	Brussels chicory juice	Phenolic acids, flavonoids and terpenes	100 g of Brussels chicory mixed with 180 mL of fresh milk	Once daily	A 7-day intervention with at least a 2-week washout period	Day 7: juice/placebo with standardized breakfast; exercise 90 min later	Usual diet/activity; exercise, caffeine, energy drinks, alcohol avoided 48 h pre-test.	NR
Salem et al. [42]	Beetroot	Bio beetroot powder	Nitrate	15 g powder per day	Daily during intervention	3 consecutive days	2 h before laboratory testing sessions	Nitrate-rich foods, stimulants, gum, alcohol, oral-hygiene products restricted	NR
Parklak et al. [43]	Nam Dok Mai mango	Mango puree	Carbohydrates and mango phytochemicals; specific bioactive NR	600 g/day, divided into three 200 g servings	Three servings/day	4 weeks per condition; 2-week washout	One serving 1 h before morning training, one during morning training, and one 1 h before evening training;	Three consecutive 24-h dietary recalls	NR
Juniarsana et al. [44]	Banana, honey, tomato	Pismatom juice	Carbohydrate, potassium-related food matrix; specific bioactive NR	240 cc per serving, containing 100 g banana, 10 g honey, 100 g tomato, and 30 cc water	Before and after physical activity	14 days; post-test measurement on day 15	30 min before and 30 min after exercise/physical activity	NR	NR
Daneshparvar et al. [45]	Beetroot	Beetroot juice	Dietary nitrate and beetroot phytochemicals	70 mL beetroot juice, providing approximately 400 mg nitrate	Single acute dose	Acute intervention during climbing session	2.5 h before ascending Riz o Boland Mountain to 3720 m	NR	NR
Toscano et al. [46]	purple grape juice	Purple grape juice	Polyphenols and antioxidant compounds	10 mL/kg body weight; 200 mL contained approximately 130 kcal and 32 g carbohydrate	Single dose per condition	Acute crossover trial; two experimental sessions separated by 1-week washout	2 h before the running test to exhaustion at 80%	Avoided exercise for 48 h, fasted overnight for 10 h, avoided nutritional supplements and antioxidant foods rich in grape polyphenols during the study	NR
Wangdi et al. [47]	Montmorency tart cherry	Montmorency cherry concentrate	Polyphenolics and anthocyanins	2 × 30 mL/day; total 60 mL/day	Twice daily, morning and evening	10 days per trial: 7 days before exercise and continued for 48 h after exercise; 2-week washout	Morning and evening	maintained normal diet but avoided increasing high-polyphenol foods during supplementation and washout	NR
Hunt et al. [49]	New Zealand blackcurrant	Anthocyanin-rich blackcurrant extract capsule	Anthocyanins, mainly delphinidin-3-rutinoside, delphinidin-3-glucoside, cyanidin-3-rutinoside, and cyanidin-3-glucoside	3 g extract/day; one 300 mg active cassis capsule containing 105 mg anthocyanins	Once daily	12 days: 8 days before exercise and 4 days after exercise	Each morning; on exercise day, 1 h before the EIMD protocol	6-day food diary; overnight fasting before testing; abstained from exercise and alcohol; NSAIDs restricted; anthocyanin intake estimated	NR
Bell et al. [48]	Montmorency tart cherry (*Prunus cerasus* L.)	Commercial tart cherry concentrate diluted with 100 mL water	Anthocyanins and total phenolic compounds; cyanidin-3-glucoside reported	30 mL per serving; 60 mL/day	Twice daily	8 consecutive days, initiated 4 days before exercise and continued throughout the 72-h recovery period	08:00 and 18:00	Low-polyphenol diet before and throughout supplementation; food diaries; avoidance of additional exercise	NR
Clifford et al., 2016 [33]	Beetroot	Beetroot juice	Nitrate, phenolics and betalains	High dose: 250 mL; low dose: 125 mL diluted to 250 mL	Three doses on day 1; twice daily at 24 and 48 h	Seven servings over 3 days after bout 1	Immediately and 2 h post-exercise and with the evening meal; morning and evening at 24 and 48 h	High-phytochemical, nitrate- and nitrite-rich foods restricted; dietary intake recorded	NR
Clifford et al., 2017 [29]	Beetroot	Beetroot juice	Nitrate, betanin and polyphenols	250 mL/serving; approximately 210 mg nitrate	Three doses on day 1; twice daily at 24 and 48 h	Seven servings over 3 days	At 30 min and 2.5 h post-exercise and with the evening meal; subsequent morning and evening doses	Usual diet recorded; standardized evening meals; overnight fasting; antibacterial mouthwash prohibited	NR
Nieman et al. [50]	Hemp	Hemp hull fiber bars	Hemp hull fiber; N-trans-caffeoyl tyramine (NCT); N-trans-feruloyl tyramine (NFT); phenolic compounds	High dose: 20 g/day hemp hull powder; low dose: 5 g/day; placebo: 0 g/day	Twice daily	2 weeks per condition, with 2-week washout periods	One bar with the first meal in the morning and one with the last meal of the day; one bar consumed before the exercise trial	3-day moderate-carbohydrate diet; high-fat foods, visible fats, caffeine and strong spices restricted; 3-day food records	No significant differences in gastrointestinal or other recorded symptoms
Rezaei et al. [51]	Pomegranate	Natural pomegranate juice	Polyphenols, including tannic acid, chlorogenic acid, ellagic acid, gallic acid, rutin and other phenolic acids	1000 mL total: 500 mL + 500 mL; total polyphenols **≈** 1744.72 mg/L	Two doses per condition	Acute supplementation surrounding EIMD; 7-day washout between conditions	500 mL evening before EIMD + 500 mL 2 h pre-EIMD	Standardized breakfast; habitual diet maintained; polyphenol-rich foods/beverages, caffeine, nitrate-rich vegetables and alcohol restricted for 48 h pretesting	NR
Garnacho-Castaño et al. [52]	Beetroot	Concentrated beetroot juice	Dietary nitrate; nitrate–nitrite–NO pathway	140 mL, providing ~12.8 mmol nitrate	Single dose per condition	Acute crossover; 1-week washout	3 h before resistance-exercise test	Diet standardized for 48 h and replicated; nitrate-rich foods restricted ≥72 h; caffeine, alcohol and other supplements prohibited; oral antibacterial products restricted	NR
Cesanelli et al. [53]	Broccoli	Freeze-dried glucoraphanin-rich broccoli powder reconstituted in hot water	Glucoraphanin (sulforaphane precursor); broccoli-derived phytochemicals	10 g broccoli powder, providing 320 µg glucoraphanin per serving	Once daily	6 days pre-exercise + test day + 4 days post-exercise	Once daily; on testing day 3 h before eccentric exercise	Regular diet maintained; participants abstained from exercise 1 week before testing and additional physical activity during experimental period; habitual cruciferous-vegetable intake assessed	NR

Intervention characteristics are presented as reported in the original studies. Where information was unavailable, it is indicated as NR: Not reported. AVA, avenanthramide; BTJ, beetroot juice; EIMD, exercise-induced muscle damage; NO, nitric oxide; NSAIDs, non-steroidal anti-inflammatory drugs; PLA, placebo; RST, repeated-sprint test; SN, sodium nitrate; TEAC, Trolox-equivalent antioxidant capacity.

## Data Availability

Data extracted from the included studies are presented in Table 1 and Table 2 and Supplementary Tables S1 and S2. Additional extracted data and risk-of-bias assessments are available from the corresponding author upon reasonable request.

## Supplementary Materials

The following supporting information can be downloaded at: https://www.mdpi.com/article/10.3390/nu18183049/s1, Table S1: Complete search strategies for Web of Science, Scopus, Embase, and PubMed; Table S2: Preclinical evidence; PRISMA 2020 checklist. References [58,59,60,61,66,67,73,74,75,76,77,78,79,80,81,82] is cited in the Supplementary Materials.
