# Supplementary material for: Plant-Derived Whole Foods and Food-Based Supplements on Post-Exercise Recovery in Physically Active Individuals: A Systematic Review of Human Intervention Trials
Source: Nutrients. 2026 Sep 18;18(18):3049. doi: 10.3390/nu18183049 (PMC13610232; doi:10.3390/nu18183049)
Supplement: Supplementary file 1 [file nutrients-18-03049-s001.zip › nutrients-4490208-supplementary.pdf]

Supplementary Table S1. Complete search strategies for Web of Science, Scopus, Embase, and PubMed

Database	Search strategy
Web of science	TS=("exercise recovery" OR recover* OR "muscle soreness" OR DOMS OR "muscle damage" OR fatigu* OR "perceived recovery" OR "exercise-induced muscle damage") AND TS=("plant-derived" OR "plant derived" OR "plant-based" OR "plant based" OR "plant food*" OR "whole food*" OR fruit* OR vegetable* OR juice* OR concentrate* OR powder* OR beetroot OR beet OR "tart cherry" OR cherry OR pomegranate OR banana OR watermelon OR oat* OR "whole grain*" OR soy OR "soy food*" OR tofu OR tempeh OR edamame OR mushroom* OR berr* OR grape* OR honey OR mango OR chicory) AND TS=(exercise OR athlete* OR sport* OR trained OR "physically active" OR "recreationally active") AND TS=(randomized OR randomised OR RCT OR "controlled trial" OR placebo)
Scopus	TITLE-ABS-KEY("exercise recovery" OR recover* OR "muscle soreness" OR DOMS OR "muscle damage" OR fatigu* OR "perceived recovery" OR "exercise-induced muscle damage") AND TITLE-ABS-KEY("plant-derived" OR "plant derived" OR "plant-based" OR "plant based" OR "plant food*" OR "whole food*" OR fruit* OR vegetable* OR juice* OR concentrate* OR powder* OR beetroot OR beet OR "tart cherry" OR cherry OR pomegranate OR banana OR watermelon OR oat* OR "whole grain*" OR soy OR "soy food*" OR tofu OR tempeh OR edamame OR mushroom* OR berr* OR grape* OR honey OR mango OR chicory) AND TITLE-ABS-KEY(exercise OR athlete* OR sport* OR trained OR "physically active" OR "recreationally active") AND TITLE-ABS-KEY(randomized OR randomised OR RCT OR "controlled trial" OR placebo)
Embase	('exercise recovery':ti,ab,kw OR recover*:ti,ab,kw OR 'muscle soreness':ti,ab,kw OR doms:ti,ab,kw OR 'muscle damage':ti,ab,kw OR fatigu*:ti,ab,kw OR 'perceived recovery':ti,ab,kw OR 'exercise induced muscle damage':ti,ab,kw) AND ('plant derived':ti,ab,kw OR 'plant based':ti,ab,kw OR 'plant food*':ti,ab,kw OR 'whole food*':ti,ab,kw OR fruit*:ti,ab,kw OR vegetable*:ti,ab,kw OR juice*:ti,ab,kw OR concentrate*:ti,ab,kw OR powder*:ti,ab,kw OR beetroot:ti,ab,kw OR beet:ti,ab,kw OR 'tart cherry':ti,ab,kw OR cherry:ti,ab,kw OR pomegranate:ti,ab,kw OR banana:ti,ab,kw OR watermelon:ti,ab,kw OR oat*:ti,ab,kw OR 'whole grain*':ti,ab,kw OR soy:ti,ab,kw OR 'soy food*':ti,ab,kw OR tofu:ti,ab,kw OR tempeh:ti,ab,kw OR edamame:ti,ab,kw OR mushroom*:ti,ab,kw OR berr*:ti,ab,kw OR grape*:ti,ab,kw OR honey:ti,ab,kw OR mango:ti,ab,kw OR chicory:ti,ab,kw) AND (exercise:ti,ab,kw OR athlete*:ti,ab,kw OR sport*:ti,ab,kw OR trained:ti,ab,kw OR 'physically active':ti,ab,kw OR 'recreationally active':ti,ab,kw) AND (randomized:ti,ab,kw OR randomised:ti,ab,kw OR 'randomized controlled trial':ti,ab,kw OR 'controlled clinical trial':ti,ab,kw OR rct:ti,ab,kw OR placebo:ti,ab,kw)
Pubmed	(("Exercise"[Mesh] OR exercise[Title/Abstract] OR athlete*[Title/Abstract] OR sport*[Title/Abstract] OR trained[Title/Abstract] OR "physically active"[Title/Abstract] OR "recreationally active"[Title/Abstract]) AND ("exercise recovery"[Title/Abstract] OR recover*[Title/Abstract] OR "muscle soreness"[Title/Abstract] OR DOMS[Title/Abstract] OR "muscle damage"[Title/Abstract] OR fatigu*[Title/Abstract] OR "perceived recovery"[Title/Abstract] OR "exercise-induced muscle damage"[Title/Abstract]) AND (("Plants"[Mesh] OR "Food"[Mesh] OR "Dietary Supplements"[Mesh] OR "Fruit"[Mesh] OR "Vegetables"[Mesh]) OR "plant-derived"[Title/Abstract] OR "plant derived"[Title/Abstract] OR "plant-based"[Title/Abstract] OR "plant based"[Title/Abstract] OR "plant food"[Title/Abstract] OR "plant foods"[Title/Abstract] OR "whole food"[Title/Abstract] OR "whole foods"[Title/Abstract] OR fruit*[Title/Abstract] OR vegetable*[Title/Abstract] OR

	juice*[Title/Abstract] OR concentrate*[Title/Abstract] OR powder*[Title/Abstract] OR beetroot[Title/Abstract] OR beet[Title/Abstract] OR "tart cherry"[Title/Abstract] OR cherry[Title/Abstract] OR pomegranate[Title/Abstract] OR banana[Title/Abstract] OR watermelon[Title/Abstract] OR oat*[Title/Abstract] OR "whole grain"[Title/Abstract] OR "whole grains"[Title/Abstract] OR soy[Title/Abstract] OR "soy food"[Title/Abstract] OR "soy foods"[Title/Abstract] OR tofu[Title/Abstract] OR tempeh[Title/Abstract] OR edamame[Title/Abstract] OR mushroom*[Title/Abstract] OR berr*[Title/Abstract] OR grape*[Title/Abstract] OR honey[Title/Abstract] OR mango[Title/Abstract] OR chicory[Title/Abstract]) AND (randomized[Title/Abstract] OR randomised[Title/Abstract] OR RCT[Title/Abstract] OR "controlled trial"[Title/Abstract] OR placebo[Title/Abstract] OR randomized controlled trial[Publication Type] OR controlled clinical trial[Publication Type]))

Supplementary Table S2. Preclinical mechanistic evidence: Selected cell-based and animal evidence supporting potential mechanisms of plant-derived foods and plant-derived bioactive compounds relevant to exercise recovery

Author / Year	Model Type	Bioactive / Supplement source	Dose	Mechanistic findings	Relevance to human recovery
Zhang et al., 2024 [67]	Equine skeletal muscle satellite cells exposed to 600 μM H_2O_2 for 6 h.	Mulberry leaf flavonoids	0.8 mg/mL CEP, 0.6 mg/mL RP, and 0.6 mg/mL NB-EP.	Improved cell viability; increased SOD, GSH-Px, T-AOC and Nrf2-related genes; reduced MDA; enhanced mitochondrial respiration, ATP production and glycolytic capacity.	Supports antioxidant and mitochondrial mechanisms that may preserve muscle satellite-cell survival and regeneration after exercise-related oxidative stress. Muscle function was not directly assessed.
Cuijpers et al., 2024[74]	C2C12 mouse myoblasts differentiated	Hesperetin	Hesperetin 5–50 μM; ellagic acid 0.05–0.1 μM; menadione 9 μM	At 20 μM, ↑ myosin heavy-chain expression and myoblast fusion	Suggests promotion of myogenic differentiation and myotube formation during oxidative stress.
Sahin et al., 2016 [58]	Male Wistar rats	Curcumin	CurcuWIN® 100 mg/kg/day for 6 weeks;	↓ lactate, muscle MDA, NF-κB and HSP70; ↑ SOD, GPx, GSH, Nrf2/HO-1, TRX-1, SIRT1, PGC-1α and GLUT4; improved time to exhaustion	Supports antioxidant, anti-inflammatory and mitochondrial mechanisms that may protect skeletal muscle during intensive exercise.
Park et al., 2025 [61]	Male ICR mice with methylglyoxal-induced skeletal-muscle atrophy undergoing treadmill exercise	trans-Resveratrol and hesperidin	200 mg/kg/day for 8 weeks: 70 mg/kg resveratrol + 130 mg/kg hesperidin; treadmill 5 days/week	↑ AMPK/SIRT1/PGC-1α, AKT/mTOR, MyoD and MyHC; ↓ MuRF1 and IL-6; ↑ IL-10; reduced fibrosis; increased muscle mass, fiber CSA, grip strength and endurance	Provides strong mechanistic evidence for preservation of muscle mass and function through mitochondrial biogenesis, protein synthesis, reduced proteolysis, myogenesis and anti-fibrotic effects. Relevance

Author / Year	Model Type	Bioactive / Supplement source	Dose	Mechanistic findings	Relevance to human recovery
					to post-exercise recovery is indirect because the model represents metabolic-stress-induced atrophy rather than exercise-induced muscle damage.
Yu et al., 2025[75]	Primary mouse skeletal muscle cells: mechanical stretch; C57BL/6 mice	Gallic acid	Cells: 50 μM; mice: 200 mg/kg/day	↓ CK, LDH, IL-6, TNF-α, ROS, MDA, lipid peroxidation and ferroptosis-related markers; ↑ GPX4, SOD, GSH/GSSG, ATP and mitochondrial integrity	Provides direct mechanistic evidence for protection against exercise-related muscle damage through inhibition of oxidative stress and ferroptosis.
Yamamoto et al., 2022 [76]	Primary rat skeletal-muscle satellite cells, L6 and C2C12 myoblasts	Lemon myrtle	Cells: LM 1–10 μg/mL; casuarinin 50–400 nM. Rats: LM 250 mg/kg/day or casuarinin 4 or 8 mg/kg/day for 4 days	↑ satellite-cell proliferation, IL-6 expression and satellite-cell activation	Supports satellite-cell activation as a potential mechanism for muscle repair and regeneration.
H. Lee et al., 2025 [77]	TNF-α-treated differentiated L6 myotubes; male C57BL/6N mice	Polyphenol-rich *Rosa canina* fruit extract	Cells: 40 or 80 μg/mL for 24 h. Mice: 200 or 400 mg/kg/day orally for 1 week after 1 week of immobilization	↓ TNF-α/IL-6/NF-κB, myostatin, MuRF1 and Atrogin-1; ↑ antioxidant enzymes, MyoD, myogenin, MyHC and PI3K/Akt/mTOR signaling; preserved muscle mass, fiber CSA and volume; improved grip strength and endurance	Provides strong supportive evidence for anti-inflammatory, antioxidant, anabolic and anti-proteolytic mechanisms underlying muscle-mass and functional recovery.
Rusip et al., 2022 [78]	Male rats	Red dragon-fruit extract	75, 150 or 300 mg/kg/day	↓ blood MDA; ↑ SOD; reduced soleus-muscle inflammation and necrosis; prolonged swimming duration,	Provides exercise-related evidence for antioxidant and tissue-protective effects of a polyphenol-

**Author / Year**	**Model Type**	**Bioactive / Supplement source**	**Dose**	**Mechanistic findings**	**Relevance to human recovery**
				particularly at higher doses	rich fruit extract; molecular pathways and post-exercise force recovery were not assessed.
Liu et al, 2022[60]	Male BALB/c mice (3 months of age)	Lonicera caerulea berry extract	250 mg/kg	↑ AMPK–PGC-1α and IGF-1–PI3K/Akt/mTOR signaling, ATP, glycogen, SOD and GSH-Px; ↓ lactate, MDA, inflammation and apoptosis	Suggests improved energy metabolism, reduced oxidative stress and protection against prolonged exercise fatigue.
Song et al, 2025 [79]	30 male C57BL/6J mice	Icariin (ICA)	ICA (25 or 50 mg/kg) via daily gavage for 8 consecutive weeks	↓ MDA, ↑ SOD, ↑ CAT, ↑ GSH-PX, ↓ TNF-α, ↓ NF-κB, ↓ IL-6, ↓ IL-1β, ↓ LDH, ↓ BUN, ↓ CK	Protects against exercise-induced acute muscle damage and systemic inflammation, facilitating faster recovery after exhaustive exertion
Yada et al, 2018 [59]	Seventy-eight male C57BL/6J mice	Taheebo Polyphenol	200 mg/kg body weight	↑ running time, ↑ blood glucose, ↑ muscle glycogen, ↓ protein carbonyls, ↑ p-AMPK, ↑ SIRT1	Enhances endurance capacity and energy substrate availability, potentially speeding up glycogen replenishment after exercise
Yada et al, 2019 [80]	Twenty-four 9-week-old male C57BL/6J mice	Acacia Polyphenol (AP)	High dose AP 200 mg/kg body weight	↓ Skeletal muscle TBARS (lipid peroxidation)	Acts as a specialized scavenger for muscle-specific oxidative stress. However, human translation requires caution regarding liver safety, as high doses showed markers of

Author / Year	Model Type	Bioactive / Supplement source	Dose	Mechanistic findings	Relevance to human recovery
					hepatotoxicity in this model.
Yoshida et al 2018 [81]	9 weeks old male Wistar rats	Apple Polyphenols (AP)	0.5–5% AP in diet	↓ TSP-1, ↑ MyHC I, ↑ MyHC IIa, ↑ MyHC IIx, ↓ MyHC IIb, ↑ PECAM-1 density (angiogenesis), ↑ PECAM-1/fiber ratio	Provides indirect evidence that improved muscle vascularization may support oxygen and nutrient delivery during recovery
Yoshida et al, 2023 [66]	male Wistar rats	Apple Polyphenols (AP)	0.5–5% AP in diet	↑ TFEB nuclear translocation, ↑ Complex II activity, ↑ Complex II+III activity, ↑ Complex IV activity, ↑ LC3B-II, ↑ Citrate synthase activity	Improves energy production efficiency and maintains mitochondrial quality, essential for long-term muscle health and endurance
Yeo et al. 2019 [82]	TNF-α-treated C2C12 myotubes	Avenanthramides A, B, and C—oat-derived phytochemicals	AVA-A, -B, or -C: 30 μM for 24 h; TNF-α: 10 ng/mL	↓ NF-κB activation, IL-6, IL-1β, ROS, Atrogin-1 and MuRF1; ↑ SOD2; preserved cell viability and myotube diameter	Supports anti-inflammatory, antioxidant, and anti-proteolytic mechanisms potentially relevant to oat-based recovery interventions

This table includes selected cell-based and animal studies used solely to support the interpretation of potential biological mechanisms. These studies were not included in the PRISMA study-selection process, efficacy synthesis, or risk-of-bias assessment, and their findings should not be interpreted as direct evidence of effectiveness in humans. ↑ indicates an increase or activation; ↓ indicates a decrease or inhibition. AKT, protein kinase B; AMPK, AMP-activated protein kinase; ATP, adenosine triphosphate; BUN, blood urea nitrogen; CAT, catalase; CK, creatine kinase; COX-2, cyclooxygenase-2; CSA, cross-sectional area; GPx/GSH-Px, glutathione peroxidase; GLUT4, glucose transporter type 4; IL, interleukin; LDH, lactate dehydrogenase; MDA, malondialdehyde; mTOR, mechanistic target of rapamycin; MuRF1, muscle RING-finger protein 1; MyHC, myosin heavy chain; MyoD, myogenic differentiation protein; NF-κB, nuclear factor kappa B; Nrf2, nuclear factor erythroid 2-related factor 2; OXPHOS, oxidative phosphorylation; PGC-1α, peroxisome proliferator-activated receptor-γ coactivator-1α; PI3K, phosphoinositide 3-kinase; ROS, reactive oxygen species; SIRT1, sirtuin 1; SOD, superoxide dismutase; T-AOC, total antioxidant capacity; TFEB, transcription factor EB; TNF-α, tumor necrosis factor alpha; VO_2max, maximal oxygen uptake.
